# Erythroside: A New Cerebroside and Other Compounds From *Erythrina Caffra* Thunb. (Fabaceae) Stem Bark, With Cytotoxicity and Antioxidant Evaluation

**DOI:** 10.1002/cbdv.202503577

**Published:** 2026-04-09

**Authors:** Bienvenu Tsakem, June C. Serem, Yvette N. Hlophe, Michael H. Kamdem Kengne, Louis P. Sandjo, Derek T. Ndinteh, Rémy B. Teponno, Melvin A. Ambele, Xavier Siwe Noundou

**Affiliations:** ^1^ Department of Pharmaceutical Sciences School of Pharmacy Sefako Makgatho Health Sciences University Pretoria South Africa; ^2^ Department of Anatomy School of Medicine University of Pretoria Pretoria South Africa; ^3^ Department of Physiology School of Medicine University of Pretoria Pretoria South Africa; ^4^ Centre For Natural Product Research (CNPR), Department of Chemical Sciences University of Johannesburg Johannesburg South Africa; ^5^ Drug Discovery and Smart Molecules Research Laboratory, Department of Chemical Sciences University of Johannesburg Johannesburg South Africa; ^6^ Department of Chemistry, Federal University of Santa Catarina Campus Universitário‐Trindade Florianópolis Santa Catarina Brazil; ^7^ Department of Chemistry Faculty of Science University of Dschang Dschang Cameroon; ^8^ Department of Oral and Maxillofacial Pathology, Faculty of Health Sciences University of Pretoria Pretoria South Africa

**Keywords:** ABTS, Cerebroside, cytotoxicity, *Erythrina caffra*, ORAC

## Abstract

The chemical investigation of a CH_2_Cl_2_:MeOH (1:1) extract of the stem bark of *Erythrina caffra* resulted in the isolation and characterisation of the novel cerebroside, erythroside (**1**), alongside eight other known compounds (**2‐9**). The isolated compounds were characterized by FT‐IR, 1D and 2D NMR spectroscopy, and ESI‐MS analysis. Of the eight known compounds, **2**, **3**, **5,** and **7** are isolated for the first time from the genus *Erythrina*. The crude extract, its fractions, and selected isolated compounds were evaluated for cytotoxicity against three normal cell lines, human keratinocytes (HaCaT), human melanocytes (NHEM‐Ad), and Human Embryonic Kidney 293 (HEK293), using the resazurin/Alamar blue and crystal violet assays. Antioxidant potential was also assessed through both the oxygen radical absorbance capacity (ORAC) and the Trolox equivalent antioxidant capacity (TEAC) assays. Most compounds showed no cytotoxic effects on these cell lines. The antioxidant assay revealed that compound **1** had a moderate antioxidant effect in the ABTS assay (0.50 µmol TE/mg) and good antioxidant capacity in the ORAC assay (0.87 µmol TE/mg). In contrast, almost all these compounds and extracts have shown moderate to good antioxidant effects in the ORAC assay. The results offer valuable insights into the chemical constituents of *Erythrina caffra* stem bark, their effects on HaCaT, NHEM‐Ad, and HEK 293 cells, as well as their antioxidant potential. Future research could focus on identifying additional bioactive compounds within the ethyl acetate fraction that may be responsible for the observed toxicity.

## 1 Introduction

Natural products constitute an important source of structurally diverse bioactive compounds with considerable therapeutic potential. In fact, more than 60% of today's anticancer drugs originate wholly or partially from natural sources, underscoring their central role in the development of new treatments [1, 2]. Among them, secondary metabolites such as alkaloids, terpenoids, flavonoids, and glycosphingolipids play essential roles in mediating pharmacological responses, including antioxidant, anti‐inflammatory, and anticancer activities [3, 4].

*Erythrina caffra* Thunb. belongs to the Fabaceae family. This plant is indigenous to southeastern Africa [5] and in South Africa, where it is mainly located in the warm and frost‐free to light frost regions [6]. It is attractive for its warm red to scarlet‐coloured flowers, which commonly appear from the cold winter months (May‐July) up to spring (August‐October) before the leaves [5]. Its trunk and branches are grey, sometimes set with short, sharp prickles [7]. The previous chemical studies of its stem bark led to the isolation of several prenylated flavonoids and other phenolic compounds [6, 8]. As part of the ongoing search for secondary metabolites from African medicinal plants with potent biological benefits [9], the present study focused on the stem bark of *Erythrina caffra*, from which nine compounds were successfully isolated and characterised.

## 2 Results

### 2.1 Structure Elucidation and Identification of Isolated Compounds

**FIGURE 1:**
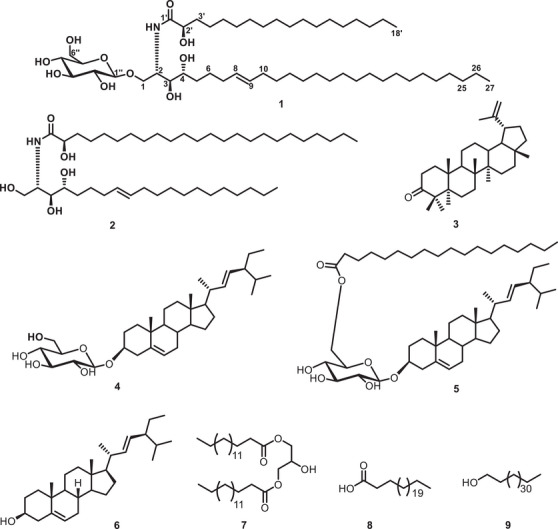
Chemical structures of compounds **1‐9**.

The CH_2_Cl_2_:MeOH (1:1) extract of *Erythrina caffra* stem bark was subjected to column chromatography over silica gel and Sephadex LH‐20 as stationary phases to afford a previously unreported cerebroside (**1**), (2*S*,3*S*,4*R*,8*E*)‐2‐[(*R*)‐2'‐hydroxytetracosanoylamino]‐8‐en‐1,3,4‐eicosenetriol (**2**) [10], lupen‐3‐one/moretenone (**3**) [11, 12], stigmasterol 3‐*O*‐*β*‐D‐glucopyranoside (**4**) [9], stigmasterol 3‐*O*‐*β*‐D‐glucopyranoside 6'‐hexadecanoate (**5**) [13], stigmasterol (**6**) [5], 1,3‐dipalmitin (**7**) [14], tetracosanoic acid (**8**) [15] and octacosan‐1‐ol (**9**) [16] (Figure 1).

Compound **1** was obtained as a yellow amorphous solid. Its FTIR spectrum (Figure S1) exhibited some characteristic bands at ν_max_ = 1560 and 1620 cm^−1^ characterising the amide function (Csp2‐N), 2850, 2950 cm^−1^ for Csp2‐H, and 3100–3600 cm^−1^ typical of OH groups. Its HRESIMS (+) (Figure S2) showed the protonated molecular ion [M+H]^+^ at m/z 886.7346 (calcd for C_51_H_100_O_10_N^+^: 886.7342), corresponding to the molecular formula C_51_H_99_O_10_N, with 3 unsaturations.

The ^1^H NMR spectrum (Figure S3) of compound **1** exhibited a resonance at *δ*_H_ 0.87 (H‐27, H‐18', s, 2CH_3_), and a range of signals at *δ*_H_ 1.20‐1.31 (H‐5 to H‐15; H‐12 to H‐24) characterizing two fatty acid moieties. The signals observed at *δ*_H_ 3.67 (o, H‐1b), 3.82 (m, H‐1a), 3.40 (o, H‐3), 3.35 (m, H‐4), 3.86 (dq, 11.3, 4.2, H‐2'), 4.15 (d, *J* = 7.8 Hz, H‐1′′), 2.95 (td, *J* = 8.4, 3.4 Hz, H‐2''), 3.10 (td, *J* = 6.7, 5.7 Hz, H‐3′′), 3.05 (dt, *J* = 9.3, 4.7 Hz, H‐4′′), 3.15 (td, *J* = 8.9, 3.9 Hz, H‐5′′), 3.67 (o, H‐6′′a), and 3.45 (dt, *J* = 11.4, 5.6 Hz, H‐6′′b) are attributed to oxymethine and oxymethylene protons. From ^13^C NMR and DEPT spectra (Figures S4,S5), the signals at *δ*_C_ 103.9 (C‐1′′), 73.9 (C‐2′′), 77.0 (C‐3′′), 70.6 (C‐4′′), 77.4 (C‐5′′), and 61.6 (C‐6′′) are attributed to *β*‐D‐glucopyranosyl moiety [17]. The configuration of the sugar moiety was determined to belong to the D‐series. This assignment was supported by the diagnostic chemical shift values, which are consistent with those reported by Agrawal (1992) [18]. In addition, this D‐configuration is also the most encountered in plant kingdom according to previous reports [19]. The other signals exhibited at *δ*_C_ 69.3 (C‐1), 50.4 (C‐2), 74.6 (C‐3), 71.1 (C‐4), 174.0 (C‐1'), and 71.4 (C‐2') are characteristic of ceramides [20, 21]. This assertion was supported by the correlation observed on the HSQC spectrum (Figure S6) from the proton at *δ*_H_ 4.10 (H‐2) to the carbon at *δ*_C_ 50.4 (C‐2) corresponding to an azomethine. The correlation from the proton at *δ*_H_ 5.37 (o, H‐8/H‐9) to the carbon at 130.7 (C‐8) and 130.1 (C‐9) indicated the presence of one double bond in compound **1**, and its *E* configuration was evidenced from the chemical shifts of the neighbouring carbons displayed at *δ*_C_ 32.5 (C‐7) and 32.7 (C‐10) [22].

**FIGURE 2:**
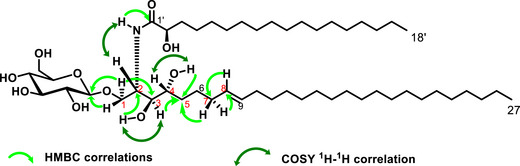
Some HMBC and COSY correlations of compound **1**.

The HMBC spectrum (Figure S7) permitted to justify the connectivity of some carbons. The correlations from the proton at *δ*_H_ 3.82 (H‐1a), and 3.67 (H‐1b) to the carbons at *δ*_C_ 103.9 (C‐1′′), 50.4 (C‐2), and 74.6 (C‐3) supported the fixation of *β*‐D‐glucopyranosyl at C‐1. On the other hand, the correlation from the proton at 7.52 (2‐NH) to the carbons at 50.4 (C‐2) and 174.0 (C‐1) confirmed that compound **1** is a cerebroside. However, the position of the double bond was located through several HMBC correlations, the correlation from the proton at 1.93 (H‐7) to the carbons at 130.7 (C‐8), 130.1 (C‐9), 26.0 (C‐6), 32.2 (C‐5); then the correlation from the proton at 3.40 (H‐4), and 4.30 (4‐OH) to the carbon at 32.2 (C‐5) and finally the correlation from the proton at 1.49 (H‐6) to the carbon at 71.1 (C‐4) supported the location of the double bond at C‐8. Some important COSY ^1^H‐^1^H correlations were depicted between the protons at *δ*_H_ 7.52 (1‐NH) and 4.10 (H‐1); 3.40 (H‐3) and 4.73 (3‐OH) and finally between 3.35 (H‐4) and 4.30 (4‐OH) (Figure 2).

**FIGURE 3:**
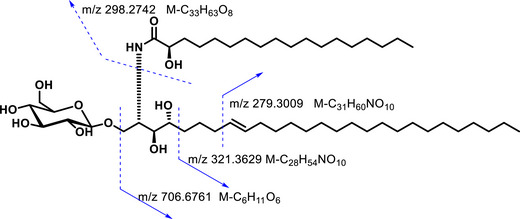
Some fragmentations from HRESIMS (+) of compound **1**.

Moreover, the length of the fatty acid chain and long chain base were supported by the fragmentation in the HRESIMS(+), the ion fragment at *m/z* 298.2742 [C_18_H_36_NO_2_]^+^ corresponded to the fatty acid chain; the fragments at m/z 279.3009 [C_20_H_39_]^+^, 321.3629 [C_23_H_45_]^+^, and 706.6761 [C_45_H_88_NO_4_]^+^ allowed to determine the length of the long chain base (Figures 3 and S9).

The stereochemistry at C‐2, C‐3, C‐4, and C‐2' were assigned 2*S*, 3*S*, 4*R* and 3'*R* from biosynthetic consideration of sphingolipids [23, 24]. Therefore, the structure of compound **1** was elucidated as 1‐*O*‐*β*‐d‐glucopyranosyl‐(2*S*,3*S*,4*R*,8*E*)‐2‐[(2‘*R*)‐2‘‐hydroxyoctadecanoylamino]‐octadec‐8‐ene‐1,3,4‐triol to which the trivial name erythroside was given. The proton NMR spectra of compounds **2‐9** are Figures S10‐S20, respectively (Supplementary material).

Though it is the first report of a cerebroside from *E. caffra*, Talla and coworkers isolated and characterised the ceramide droogmansiamide from *E. droogmansiana* [20], as for, Moretenone (**3**), stigmasterol 3‐*O*‐*β*‐D‐glucopyranoside 6'‐hexadecanoate (**5**), and 1,3‐dipalmitin (**7**), they are isolated for the first time from the genus *Erythrina*. Stigmasterol 3‐*O*‐*β*‐D‐glucopyranoside 6'‐hexadecanoate (**5**) was previously obtained from the stem bark of *Tetrapleura tetraptera* and it displayed a good activity against *Bacillus subtilis* and weak antibacterial activity against other bacteria including *Proteus mirabilis*, *Klebsiella oxytoca*, *Klebsiella aerogenes* [13], stigmasterol (**6**) and octacosan‐1‐ol (**9**) were isolated from the stem bark of *E. caffra* [5, 16]; compound **8** was isolated from the stem barks of *Erythrina addisoniae* [25].

### 2.2 Cytotoxicity Assays

The toxicity of the crude extract, ethyl acetate fraction, and compounds **1, 2, 4, 6,** and **9** was evaluated on human keratinocytes (HaCaT), human melanocytes (NHEM‐Ad), and Human Embryonic Kidney 293 (HEK 293). Selection was based on the fact that these compounds were isolated in significantly larger amounts than the others.

**FIGURE 4:**
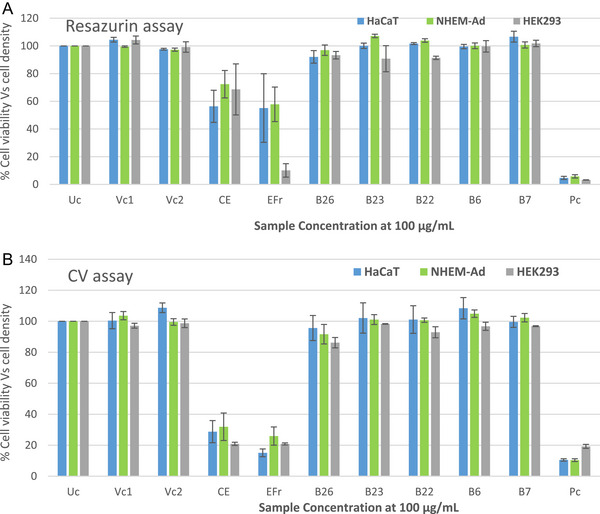
The effect of crude extract, ethyl acetate fraction and some isolated compounds on the cell number of HaCaT (blue), NHEM‐Ad (green), and HEK 293 (grey) displayed as percentage cell viability (A) and percentage cell density (B) for resazurin and Cristal violet (CV) assays, respectively. Cell number (10*10^4^), number of biological replicate n = 3. Cell number values are expressed as the percentage relative to the complete culture medium. The standard error of the mean is indicated by the error bars. (Uc = untreated cells, VC = vehicle control, CE = crude extract, EFr = ethyl acetate fraction, B26 = compound **1**, B23 = compound **2**, B22 = compound **4**, B6 = compound **6**, B7 = compound **9**, and PC = positive control which is 1% triton X 100).

Cytotoxicity results are summarised in Figure 4. According to statistical analysis using ordinary one‐way ANOVA, the majority of tested samples did not significantly affect cell viability. In the resazurin assay, compounds **1, 2, 4, 6,** and **9** consistently showed no cytotoxicity in all cell lines, however, the crude extracts (CE) and the ethyl acetate fraction (EFr) consistently showed cytotoxicity to a level of 56.38 ± 11.6 % (p = 0.0005) and 55.14 ± 24.76 % (p = 0.0012) in HaCaT cells, then 72.37 ± 9.89 % (p = 0.0069) and 57.84 ± 12.45 % (p < 0.0001) in NHEM‐Ad cells, respectively. They equally affected the viability of HEK 293 at 68.6 ± 18.42 (p = 0.0075) and 10.11 ± 4.86 (p < 0.0001). This trend was the same in the CV assay with CE and EFr consistently showed cytotoxicity to a level of 28.77 ± 7.14 and 15.09 ± 2.50 % in HaCaT cells, then 31.92 ± 8.87 and 25.91 ± 5.88 % in NHEM‐Ad cells with p < 0.0001 for each. Similarly, these two samples showed cell density of 20.89 ± 1.03, 20.84 ± 0.67 on HEK 293 with p < 0.0001. The safety profile is more or less uniform across all cell types. These findings suggest that renal and dermal tissues could be more vulnerable to some of the tested samples. The recent study by Olawale et al. demonstrated that the ethyl acetate fraction of the stem barks reduced HEK293 cell viability by approximately 50% at 100 µg/mL. A slight decrease in cell viability was observed at 150, 200, and 250 µg/mL [26]. Interestingly, the study also showed that HEK 293 cells remained viable at concentrations up to 50 µg/mL [26], suggesting that toxicity may only manifest at higher doses. This points toward a concentration‐dependent effect and indicates that therapeutic windows may exist where the compounds exert pharmacological benefits without inducing adverse cellular responses. Additionally, the fatty alcohol Heinecosanol isolated from the dichloromethane extract did not show any sign of toxicity on HEK293 [26]. This observation corroborates our findings with compound **9**, which similarly did not compromise the viability of any of the tested cell lines. Ceramide **2** exhibited no signs of toxicity at the tested concentration (141.04 µM), previous reports indicated that a related ceramide, namely (2*S*,3*S*,4*R*)‐2‐[(2*R*)‐2‐hydroxytetracosanoylamino]‐1,3,4‐octadecanetriol did not affect the viability of HepG2 cells at 200 µM [27].

The effect of the samples appeared more pronounced in the Crystal Violet assay, as only the adherent cells remaining in each well contributed to the absorbance measurement. Microscopic images (Figures S20,S21) were also captured to provide a visual confirmation of the effects in each well. A comparison between untreated cells, vehicle and positive controls, and wells treated with the samples clearly shows that the crude extract together with the ethyl acetate fraction induced significant cell death in both HaCaT and melanocyte cell lines at a concentration of 100 µg/mL. Moreover, the two complementary assays showed good consistency, with respective correlation coefficients (R^2^) of 0.8568, 0.8336, and, 0.8026 in HaCaT, NHEM‐Ad, and HEK 293 cells, respectively, thereby confirming the reliability of the cytotoxicity measurements.

### 2.3 Antioxidant Capacity

**FIGURE 5:**
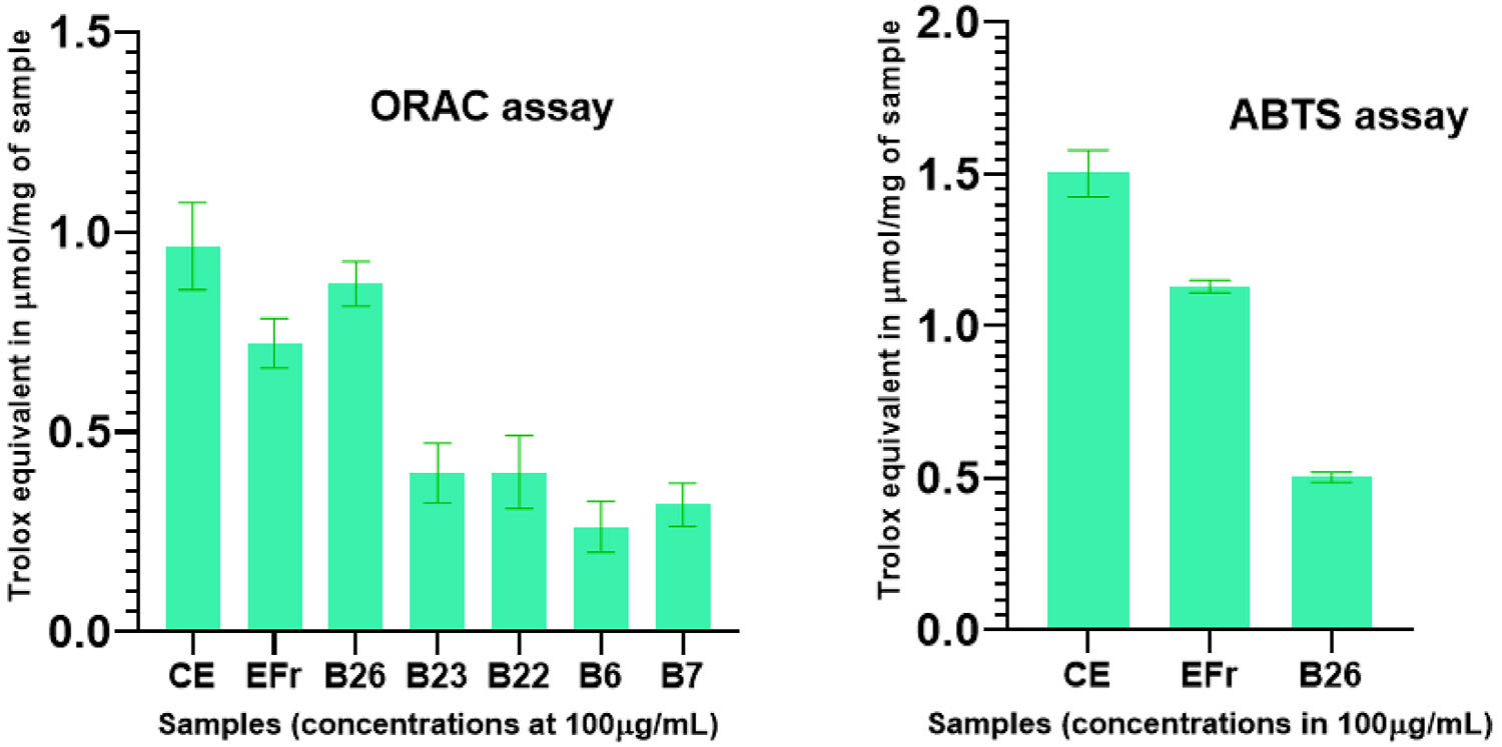
Antioxidant capacity of the crude extract, EtOAc fraction and some isolated compounds assessed both in ORAC and ABTS (sample concentrations 100 µg/mL). CE = crude extract, EFr = ethyl acetate fraction, B26 = compound **1**, B23 = compound **2**, B22 = compound **4**, B6 = compound **6**, B7 = compound **9**.

The antioxidant potential of the samples was assessed using two complementary methods: oxygen radical absorbance capacity (ORAC) and the Trolox equivalent antioxidant capacity (TEAC) assays or commonly known as ABTS assay. The results indicated that the crude extract and ethyl acetate (EtOAc) fraction exhibited antioxidant capacities of 0.96 and 1.01 µmol TE/mg, respectively, in the ORAC assay, and 1.50 and 1.13 µmol TE/mg (Figure 5, Table 2), respectively, in the ABTS assay, classified as moderate activity.

**TABLE 1:** ^1^H NMR (DMSO‐*d*_6_, 500 MHz) and ^13^C NMR (DMSO‐*d*_6_, 125 MHz) data of compound **1**.

Position	*δ*_C_ in ppm	*δ*_H_ (multiplicity, *J* in Hz, integrals) in ppm
**1**	69.3	3.67 (o, H‐1a), 3.82 (m, 1H)
**2**	50.4	4.10 (dt, 5.1, 3.7, 1H)
**3**	74.6	3.40 (o, 1H)
**4**	71.1	3.35 (m, 1H)
**5**	32.2	1.50 (m, 2H)
**6**	26.0	1.49 (m, 2H)
**7**	32.5	1.93 (o, 2H)
**8**	130.7	5.37 (o, 1H)
**9**	130.1	5.37 (o, 1H)
**10**	32.7	1.93 (o, 2H)
**11‐24**	29.0‐29.6	1.24‐1.31 (m, 28H)
**25**	31.9	1.25 (o, 2H)
**26**	22.5	1.25 (o, 2H)
**27**	14.8	0.87 (d, 6.7, 3H)
**1'**	174.0	/
**2'**	71.4	3.86 (dq, 11.3, 4.2, 1H)
**3'**	34.8	1.61 (ddt, 13.8, 9.9, 4.8, 1H); 1.49 (m, 1H)
**4'**	25.0	1.32 (m, 2H)
**5'‐15'**	29.0‐29.6	1.24‐1.31 (m, 22H)
**16'**	31.9	1.25 (o, 2H)
**17'**	22.5	1.25 (o, 2H)
**18'**	14.8	0.87 (d, 6.7, 3H)
**1''**	103.9	4.15 (d, 7.8, 1H)
**2''**	73.9	2.95 (td, 8.4, 3.4, 1H)
**3''**	77.0	3.10 (td, 6.7, 5.7, 1H)
**4''**	70.6	3.05 (dt, 9.3, 4.7, 1H)
**5''**	77.4	3.15 (td, 8.9, 3.9, 1H)
**6''**	61.6	3.67 (o, 1H), 3.45 (dt, 11.4, 5.6, 1H)
**2‐NH**	/	7.52 (d, 9.3, 1H)
**3‐OH**	/	4.73 (d, 5.7, 1H)
**4‐OH**	/	4.30 (d, 6.7, 1H)
**2'‐OH**	/	5.55 (d, 5.0, 1H)
**2"‐OH**	/	4.91 (d, 3.7, 1H)
**3"/4"‐OH**	/	4.86 (d, 4.2, 2H)
**6"‐OH**	/	4.73 (d, 5.7, 1H)

s: singlet, d: doublet, dt: doublet of triplets, ddt: doublet of doublets of triplets, t: triplet, td: triplet of doublets, dq: doublet of quartets, o = overlap, m = multiplets.

**TABLE 2:** ORAC and ABTS+ assays of extracts, fractions and isolated compounds from the stem barks of *E. caffra*.

	ORAC assay	ABTS assay
CEB	0.96 ± 0.11	1.50 ± 0.08
EEB	1.01 ± 0.02	1.13 ± 0.02
B6	0.26 ± 0.06	na
B7	0.32 ± 0.05	na
B23	0.40 ± 0.07	na
B22	0.40 ± 0.09	na
B26	0.87 ± 0.06	0.50 ± 0.02

CE = crude extract, EFr = ethyl acetate fraction, B26 = compound **1**, B23 = compound **2**, B22 = compound **4**, B6 = compound **6**, B7 = compound **9**, na = no activity.

The isolated compounds demonstrated a radical scavenging activity in the ORAC assay (0.3 −0.87 µmol TE/mg), with the highest activity comparable to that of the crude extract (0.96 µmol TE/mg) and EtOAc fraction (0.72 µmol TE/mg). Compound **1** had the highest activity in ORAC (0.87 µmol TE/mg) while its activity was lower in the ABTS assay (0.50 µmol TE/mg) (Figure 5, Table 2), tough it was notably the only isolated compound to show measurable activity in this test. This suggests that compound **1** has a unique ability among the isolates to engage in ET reactions with ABTS•^+^. Structurally, the carbohydrate moiety of compound **1** could have contributed to its higher activity both in ORAC and ABTS assays compared to compound **2** (0.4 µmol TE/mg in ORAC and no activity in ABTS).

The ORAC assay reflects a hydrogen atom transfer (HAT) reaction mechanism, which is considered the most biologically relevant mode of antioxidant action in human physiology, the assay measures the ability of antioxidants to inhibit peroxyl radical‐induced oxidation, thereby reflecting classical chain‐breaking antioxidant activity through HAT mechanisms [28]. The peroxyl radical reacts with fluorescein (fluorescent probe), resulting in the formation of a nonfluorescent product, which allows the oxidative degradation to be monitored over time. In the ABTS assay, antioxidants interact with the ABTS•^+^ radical cation via a single electron transfer (SET) mechanism, reducing it from its blue‐green chromophore form to the colourless ABTS molecule [29]. Sárközy et al. reported the antioxidant effect of two cerebrosides isolated from the edible mushroom *Meripilus giganteus* to be 2.50 and 1.81 µmol TE/mg [30]. Comparatively to compound **1**, some structural features like the position of the double bond and the chlorine atom could have contributed to that reported activity. (Figure 5)

The absence of ABTS•^+^ scavenging activity of other compounds implies a limited role in SET‐based antioxidant mechanisms. However, their performance in the ORAC assay indicates a capacity to neutralize peroxyl radicals via HAT, a mechanism more biologically relevant to lipid peroxidation processes. Together, these results underscore the distinct antioxidant mechanisms among the crude extract, fractions, and isolated compounds. As demonstrated by DFT calculations reported by Elshamy et al. (2020), the antioxidant activity of compound **1** may be attributed to the homolytic O–H bond dissociation within its aglycone moiety [31]. The presence of three hydroxyl groups in this region likely facilitates hydrogen atom donation, this strongly supports its superior antioxidant effect compared to the other compounds, and these results are consistent with those reported by Sárközy and coworkers in 2020 where cerebrosides bearing hydroxyl groups exhibited a lower activity (approximately 2 µmol TE/mg) than that observed for compound **1** [30]. Although the activity of stigmasterol 3‐*O*‐*β*‐D‐glucopyranoside (**4**) was not markedly pronounced, its glycosylated form appeared to exhibit relatively enhanced activity compared to stigmasterol (**6**), suggesting that the presence of the sugar moiety may contribute to modulating its biological effect. The table 2 below summarizes the activities of the samples both in ORAC and ABTS assays.

### 2.4 Discussion

This study demonstrates that, beyond the well‐documented flavonoids and their prenylated derivatives, the genus *Erythrina* also produces sphingolipids as part of its secondary metabolite repertoire. Prior to this investigation, only a single ceramide, droogmansiamide, had been reported from *Erythrina droogmansiana* [20]. The present findings therefore broaden the chemotaxonomic framework of the genus by identifying two additional sphingolipids, thereby underscoring the metabolic diversity within *Erythrina*. These results significantly enrich the phytochemical knowledge of the genus and further support the emerging relevance of sphingolipids as potential chemotaxonomic markers in *Erythrina* species.

Moreover, previous studies have demonstrated that cerebrosides and ceramides can suppress the accumulation of the pro‐inflammatory enzyme iNOS and downregulate COX‐2 protein expression in LPS‐stimulated RAW264.7 macrophages [32, 33], In addition, certain cerebrosides have shown protective effects on skin keratinocytes (HaCaT) against *Staphylococcus aureus*‐induced damage [34]. In this context, the occurrence of sphingolipids in *E. caffra* may partially support its traditional medicinal use, particularly in relation to pain and inflammation management.

Overall, the comparatively low toxicity of the isolated compounds relative to the crude extract and its EtOAc fraction suggests that the observed toxicity may result from synergistic interactions among multiple constituents present in the complex mixture. This indicates that the combined effects of several compounds, rather than a single bioactive molecule, likely contribute to the overall toxic profile.

Therefore, the development of a safe formulation from the stem bark of *E. caffra* should prioritize thorough and systematic fractionation to reduce the complexity of the extract. Deep fractionation would help eliminate potentially toxic synergistic combinations, allowing for the identification and selection of safer bioactive fractions or purified compounds suitable for therapeutic application.

## 3 Material and Methods

### 3.1 General Experimental and Procedures

One‐dimensional NMR (^1^H, ^1^ ^3^C NMR, and DEPT‐135) and 2D NMR (^1^H–^1^H COSY, HSQC, and HMBC) experiments were performed on a Bruker DRX‐500 spectrometer. The ^1^H NMR spectra were recorded at 400 and 500 MHz, while the ^1^ ^3^C NMR spectra were obtained at 100 and 125 MHz. For the environmental conditions, the temperature was 298 Kelvin (K), and deuterated solvents were used for analysis. The data were expressed in terms of chemical shifts (*δ*) with TMS (tetramethylsilane) being used as internal standard and the *J* (coupling constant) values were expressed in hertz (Hz). The mass spectra results were recorded in Electrospray Ionization Mass Spectrometry (ESIMS) ionization mode using a UPLC‐QTOF‐MS/MS apparatus. For the isolation of chemical constituents, silica gel 60 (Merck, 70–230 and 230–400 mesh) was used as a stationary phase for column chromatography. Sephadex LH‐20 gel was also employed as an additional stationary phase. Thin Layer Chromatography (TLC) was carried out on percolated silica gel 60 F_254_ (Merck) aluminium plates and monitored by UV‐Visible lamp multiband UV‐54/365 nm (Model UVGL‐58 Upland CA 91786, USA) and/or by spraying with 10% sulphuric acid followed by warming at 90°C. The spectroscopy data were interpreted using MestRenova, and the Liquid Chromatography–Mass Spectrometry (LC‐MS) data were analysed using MassLynx V4.2.

### 3.2 Plant Material

The plant material was collected on the campus of Sefako Makgatho Health Sciences University, Ga‐rankuwa (Zone 1), Pretoria North, in the Gauteng province, South Africa in July 2024. A sample of this plant was then identified at SANBI by Mothogoane MS to *Erythrina caffra* Thunb. in comparison to an authenticated sample deposited under the voucher number F. Olawale 1.

### 3.3 Extraction and Isolation of the Chemical Constituents

The stem bark of *E. caffra* was cut into small pieces, air‐dried, and ground to obtain 2.4 kg of powdered material. The powder was macerated three times (3 × 24 h) in 8 L of a CH_2_Cl_2_:MeOH (1:1) mixture at room temperature. The mixture was then subjected to ultrasound‐assisted extraction in an ultrasonic bath for 1 h at 30 °C. The combined filtrates were evaporated to dryness using a rotary evaporator connected to a vacuum pump, as previously reported [35], to yield 182 g of crude extract. A mass of 180 g of the crude extract was suspended in distilled water (500 mL) and extracted with ethyl acetate (1000 mL) for liquid‐liquid extraction. The ethyl acetate phase was collected 3 times and evaporated to dryness to yield the ethyl acetate fraction (121 g). The aqueous residue remaining was evaporated to dryness to yield the polar fraction (55 g).

A part of the EtOAc fraction (120 g) was adsorbed onto silica gel and then subjected to column chromatography using silica gel 60 (0.063–0.200 mm) eluted with *n*‐hexane, *n*‐hexane:CH_2_Cl_2_ (1:1), CH_2_Cl_2_, CH_2_Cl_2_:MeOH (9:1) and EtOAc 100% to afford six sub‐fractions BA (26.0 g), BB (20.0 g), BC (34 g), BD (20.0), and BE (5.8 g). Fraction B was subjected to column chromatography on silica gel, the eluent consisted of *n*‐hexane:CH_2_Cl_2_ (2:3); four sub‐fractions were obtained (BB1‐BB4). From BB2, compound **7** (2.5 mg) precipitated as a white amorphous powder. BB3 was adsorbed on his turn on silica gel and chromatographed on silica gel, eluted with *n*‐hexane:CH_2_Cl_2_ (4:1) to afford compound **3** (10.3 mg) as a colourless oil. The sub‐fraction BC was chromatographed on silica gel with a gradient *n*‐hexane:CH_2_Cl_2_ starting from *n*‐hexane: CH_2_Cl_2_ (9:1) by increasing the amount of CH_2_Cl_2_ up to 100%. Nine sub‐fractions were collected (BC1‐BC9). Further purification of the sub‐fraction BC1 on silica gel using *n*‐hexane:CH_2_Cl_2_ (75:25) to *n*‐hexane:CH_2_Cl_2_ (3:2) afforded compounds **8** (14.0 mg), and **9** (227.0 mg). Compound **6** (200.0 mg) precipitated in the sub‐fraction BC4. The sub‐fraction BD was equally chromatographed on silica gel using *n*‐hexane:CH_2_Cl_2_ (1:4) as eluent to afford 3 sub‐fractions BD1‐BD7. The sub‐fraction BD7 was chromatographed on silica gel and eluted with CH_2_Cl_2_:MeOH (95:5), yielding compound **5** (14.0 mg) as a colourless gum; compound **2** (30.0 mg) and compound **4** (27.0 mg) as white amorphous powders. The sub‐fraction BE was finally chromatographed on silica gel using CH_2_Cl_2_:MeOH (93:7) to yield 8 sub‐fractions. Compound **1** (30.0 mg) precipitated in the sub‐fraction BE8 as beige amorphous powder.

Erythroside (**1**): Yellow amorphous solid, FT‐IR spectrum ν_max_ = 1560 and 1620 cm^−1^ (C_sp2_‐N), 2850, 2950 cm^−1^ for C_sp2_‐H, 3100‐3600 cm^−1^ (OH groups). HRESIMS (+) (Figure S2) [M+H]^+^ at m/z 886.7346 (calcd for C_51_H_100_O_10_N^+^: 886.7342), NMR data (Table 1)

### 3.4 Liquid Chromatography–Mass Spectrometry Analysis of Compound 1

Compound **1** was analysed by LC–MS using a UPLC‐QTOF mass spectrometer operated in both positive and negative electrospray ionization (ESI) modes. The molecular ion mass, together with the corresponding fragment ions, was obtained and interpreted using the molecular formula generator in MassLynx v4.2

### 3.5 In Vitro Cell Viability Study

The cell lines included: Human keratinocytes (HaCaT), CELLONEX, and Catalogue number HACAT‐C (without an RRID); Normal Human Epidermal Melanocytes, adult (NHEM‐Ad), Lonza, and Catalogue number CC‐2586 (without an RRID), and Human Embryonic Kidney 293 (HEK293), ATCC, Catalogue number CRL‐1573, and RRID CVCL‐0045. They were all maintained in Dulbecco's modified essential medium (DMEM) supplemented with 10% v/v foetal calf serum and % v/v antibiotic/antimycotic mix in T75 flasks until 70%–80% confluent at 37°C, 5% CO_2_. At this confluency level, cells were detached using 2 mL of either 5% trypsin (HaCaT and NHEM‐Ad) or TrypLE Express (HEK 293). Once detached, cells were counted and plated at a volume of 90 µL, concentration 10x10^4^ cells/mL, and left overnight to attach. The next day cells were treated with 10 µL of compound at final concentrations of 0.1 mg/mL (100 µg/mL) and further incubated for 24 h. Controls included: a negative control—cells exposed to 10 µL of 0.1 M PBS, a positive control—cells exposed to 10 µL of 0.1% Triton X and vehicle controls—cells exposed to 10 µL of 0.1% dimethyl sulfoxide (DMSO) or 10 µL of 0.01% HCl in methanol. After exposure time, cells were assayed for cytotoxicity using either the resazurin or the crystal violet assays.

#### 3.5.1 Resazurin Assay

Following exposure, 11 µL of resazurin solution (dissolved in 0.1 M PBS) was added to each well for the resazurin assay. The plate was then incubated for additional 3 h for HaCaT and NHEM‐Ad cells, and 6 h for HEK 293 cells. Following incubation, the fluorescence was measured at an excitation at 544 nm then emission at 590 nm using a FLUOR microplate reader (Germany) operating with Omega software. Results were expressed in terms of percentage cell viability, compared to the negative control.

#### 3.5.2 Crystal Violet Assay

Immediately after recording fluorescence readings for the resazurin assay, the cells were fixed using 11 µL of 20% v/v formaldehyde (final concentration 2%) and incubated for 30 min. The wells were then rinsed with tap water and dried. Next, 100 µL of a 0.1% w/v crystal violet solution was added to each well, followed by an incubation period of 1 h at room temperature. The plates were then rinsed with tap water once more to remove excess CV dye, allowed to dry. Images of the cell morphology were then taken using a Zeiss microscope. After the cells were solubilized with 100 µL of 10% v/v acetic acid. Absorbance was then measured at 630 nm using a FLUOR microplate reader (Germany). And the results were expressed as percentage cell density relative to the negative control. The microscopic images were captured using the microscope.

The results of the cytotoxicity assays were tested for normality using the Shapiro‐Wilk test. As shown in the graphs (Figure S19), the data met the assumptions of normality. Therefore, an ordinary one‐way ANOVA was performed to determine the statistical significance of the effects of the samples on normal cells.

### 3.6 Antioxidant Capacity

#### 3.6.1 Oxygen Radical Absorbance Capacity Assay

The antioxidant capacity of the crude extract, the ethyl acetate fraction, and some isolated compounds was evaluated using the ORAC assay. A concentration of 8 mg/mL 2,2′‐Azobis(2‐amidinopropane) dihydrochloride (AAPH) was used as free radical initiator, which generates peroxyl radicals. Fluorescein solution was used at a final concentration of 1.39 × 10^6−^ nM. A calibration curve was established using Trolox as the standard at a range of 0‐1 mM. To run the assay, in a 96 fluorescent capable plate, 10 µL of sample at 1 mg/mL (to reach the final concentration of 50 µg/mL in well) was added to 165 µL of fluorescein solution and 25 µL of AAPH solution Fluorescence readings were recorded at an excitation of 485 nm and emission of 520 nm using FLUORstar OPTIMA plate reader (BMG Labtech, Offenburg, Germany) maintained at 37 °C over a period of 2 h. The area under the curve (AUC) for each well was calculated by integrating the fluorescence decay data using Microsoft Excel. The antioxidant capacity of each sample was determined relative to the Trolox standard curve and expressed as micromolar of Trolox equivalent per mg (µmol TE/mg).

#### 3.6.2 2,2′‐azino‐bis(3‐ethylbenzothiazoline‐6‐sulfonic acid)/TEAC Assay

The antioxidant potential of the crude extract, fractions, and isolated compounds was equally evaluated using the ABTS radical cation (TEAC) assay. Trolox standards were prepared following the same procedure used for the ORAC assay (0‐1 mM). The ABTS solution was freshly prepared 12 h prior to the assay by dissolving 0.219 g of ABTS in 5 mL of 0.1 M PBS and mixing it in equal volumes with 0.0041 g of potassium persulfate solution in 5 mL of 0.1 M PBS). This mixture was stored in the dark at room temperature for 12 h to allow the formation of the ABTS·^+^ radical.

For the assay, 10 µL of each test sample (1 mg/mL) was added to individual wells, followed by 290 µL of the ABTS·^+^ solution. Control wells contained only the ABTS solution and distilled water. The plate was then incubated in the dark at room temperature for 15 min, after which the absorbance was measured at 734 nm using a UV‐Vis spectrophotometer. Results were expressed as micromoles of Trolox equivalent antioxidant activity per milligram of sample.

### 3.7 Statistics

All experiments were conducted with a minimum of two to three technical replicates and three independent biological repeats to guarantee the reproducibility of the tests. Quantitative results were presented as mean ± standard error of the mean (SEM). Data normality was assessed using the Shapiro–Wilk test. For statistical analysis, one‐way ANOVA followed by Tukey's post hoc test was applied to datasets with a normal distribution (parametric) using Graphpad Prism 9.2.0.332. In cases where the data were nonparametric, the Kruskal–Wallis test was followed by Dunn's multiple comparisons test. A p‐value ≤ 0.05 was considered statistically significant.

## 4 Conclusion

The chemical study of the South African medicinal plant *E. caffra* furnished the unreported cerebroside (**1**), and four known compounds (2S,3S,4R,8E)‐2‐[(R)‐2'‐hydroxytetracosanoylamino]‐8‐en‐1,3,4‐eicosenetriol (**2**), Moretenone (**3**), stigmasterol 3‐O‐β‐D‐glucopyranoside 6'‐hexadecanoate (**5**), and 1,3‐dipalmitin (**7**) reported for the first time from the genus *Erythrina*. This study also provides insight into the in vitro cytotoxic effects of the crude extract and isolated compounds on HaCaT, NHEM‐Ad and HEK 293 cells. The results indicate that the crude MeOH:CH_2_Cl_2_ (1:1) extract and its ethyl acetate fraction reduced cell viability to a certain extent. Since no reduction in cell viability was observed for isolated compound, this finding is particularly significant for the prospective therapeutic use of these phytochemicals. Future research could focus on identifying additional bioactive compounds within the ethyl acetate fraction that may be responsible for the observed toxicity on HaCaT, NHEMD‐Ad, and HEK 293 cells and further evaluating their antiviral and anticancer activities.

## Author Contributions

**Bienvenu Tsakem**: conceptualisation, formal analysis, investigation, methodology, writing – original draft, writing – review and editing. **June Cheptoo Serem**: conceptualisation, methodology, supervision, data curation, and manuscript preparation. **Yvette Nkondo Hlophe**: methodology, supervision of cytotoxicity assays, data curation, and manuscript preparation. **Michael Kamdem**: spectroscopic analysis, data curation, and manuscript preparation. **Louis Pergaud Sandjo**: data curation and manuscript preparation. **Derek Tantoh Ndinteh**: supervision of spectroscopy analysis, data curation, and manuscript preparation. **Rémy Bertrand Teponno**: Structure elucidation, data curation, and manuscript preparation. **Melvin A. Ambele**: Data curation and manuscript preparation. **Xavier Siwe Noundou**: conceptualisation, formal analysis, funding acquisition, project administration, resources, supervision, validation, writing – review and editing.

## Funding

Professor Xavier Siwe Noundou kindly acknowledges a National Research Foundation (NRF) Competitive Support for Unrated Researchers (CSUR) grant, Number: SRUG2203291031; and a fifth BRICS STI Framework Programme grant award (BRICS JAF # 2021/305). The work reported herein was made possible through funding from the South African Medical Research Council through its Division of Research Capacity Development under the Research Capacity Development Initiative (RCDI) Programme awarded to Professor Xavier Siwe Noundou. The content and findings reported/ illustrated are the sole deduction, view, and responsibility of the researcher and do not reflect the official position and sentiments of the SAMRC.

## Conflicts of Interest

The authors have no competing interests to declare that are relevant to the content of this manuscript.

## Supporting information




**Supporting File 1**: cbdv71199‐sup‐0001‐SuppMat.pdf

## Data Availability

Additional data are found in the supporting information
