# Supplementary material for: Erythroside: A New Cerebroside and Other Compounds From Erythrina Caffra Thunb. (Fabaceae) Stem Bark, With Cytotoxicity and Antioxidant Evaluation
Source: Chem Biodivers. 2026 Apr 9;23:e03577. doi: 10.1002/cbdv.202503577 (PMC13066724; doi:10.1002/cbdv.202503577)
Supplement: Supplementary file 1 — Supporting File 1: cbdv71199‐sup‐0001‐SuppMat.pdf [file CBDV-23-e03577-s001.pdf]

Erythroside: A new cerebroside and other compounds from *Erythrina caffra* Thunb. (Fabaceae) stem bark, with cytotoxicity and antioxidant evaluation

Bienvenu Tsakem[a],[b],[c], June C. Serem[b], Yvette N. Hlophe[c], Michael H. Kengne Kamdem[d],[e], Louis P. Sandjo[f], Derek T. Ndinteh[d],[e], Rémy B. Teponno[g], Melvin A. Ambele[h], Xavier Siwe Noundou[a]

[a] Department of Pharmaceutical Sciences, School of Pharmacy, Sefako Makgatho Health Sciences University, PO Box 218 MEDUNSA, Pretoria, 0204, South Africa
[b] Department of Anatomy, School of Medicine, University of Pretoria, Pretoria, South Africa
[c] Department of Physiology, School of Medicine, University of Pretoria, Pretoria, South Africa
[d] Centre for Natural Product Research (CNPR), Department of Chemical Sciences, University of Johannesburg, P.O. Box 17011, Doornfontein, Johannesburg 2028, South Africa
[e] Drug Discovery and Smart Molecules Research Laboratory, Department of Chemical Sciences, University of Johannesburg, P.O. Box 17011, Doornfontein, Johannesburg 2028, South Africa
[f] Department of Chemistry, Federal University of Santa Catarina, Campus Universitário-Trindade, 88040-900 Florianópolis, SC, Brazil
[g] Department of Chemistry, Faculty of Science, University of Dschang, Dschang, Cameroon
[h] Institute for Cellular and Molecular Medicine, Department of Immunology, and SAMRC Extramural Unit for Stem Cell Research and Therapy, Faculty of Health Sciences, University of Pretoria, Gezina, Pretoria, 0084, South Africa

* Correspondence: Email: xavier.siwenoundou@smu.ac.za, Tel.: +27 12 521 5647 (XSN)

Abstract

The chemical investigation of a CH_2Cl_2:MeOH (1:1) extract of the stem bark of *Erythrina caffra* resulted in the isolation and characterisation of the novel cerebroside, erythroside (**1**), alongside eight other known compounds (**2-9**). The isolated compounds were characterised using spectroscopic techniques (FT-IR, 1D and 2D NMR) and spectrometric analysis (ESI-MS). Of the eight known compounds, **2**, **3**, **5,** and **7** are isolated for the first time from the genus *Erythrina*. The crude extract, its fractions, and selected isolated compounds were evaluated for cytotoxicity against three normal cell lines, human keratinocytes (HaCaT), human melanocytes (NHEM-Ad), and Human Embryonic Kidney 293 (HEK293), using the resazurin/Alamar blue and crystal violet assays. Antioxidant potential was also assessed through both the oxygen radical absorbance capacity (ORAC) and the Trolox equivalent antioxidant capacity (TEAC) assays. Most compounds showed no cytotoxic effects on these cell lines. The antioxidant assay revealed that compound **1** had a moderate antioxidant effect in the ABTS assay (0.50 µmol TE/mg) and good antioxidant capacity in the ORAC assay (0.87 µmol TE/mg). In contrast, almost all these compounds and extracts have shown moderate to good antioxidant effects in the ORAC assay. The results offer valuable insights into the chemical constituents of *Erythrina caffra* stem bark, their effects on HaCaT, NHEM-Ad, and HEK 293 cells, as well as their antioxidant potential. Future research could focus on identifying additional bioactive compounds within the ethyl acetate fraction that may be responsible for the observed toxicity.

Keywords: *Erythrina caffra*, Cerebroside, cytotoxicity, ORAC, ABTS

Supplementary material

The supplementary material includes detailed spectroscopic and analytical data supporting the structural characterization of all synthesized compounds. These include FTIR, ^{1}H and ^{13}C NMR (1D and 2D), and mass spectra, which confirm the proposed molecular structures and purity of the isolated products. Additionally, the Shapiro–Wilk normality test was performed on the cytotoxicity data obtained for the HaCaT, NHEM-Ad, and HEK293 cell lines to assess the data distribution prior to further statistical analysis. The corresponding spectra and statistical outputs are provided in the Supplementary Information

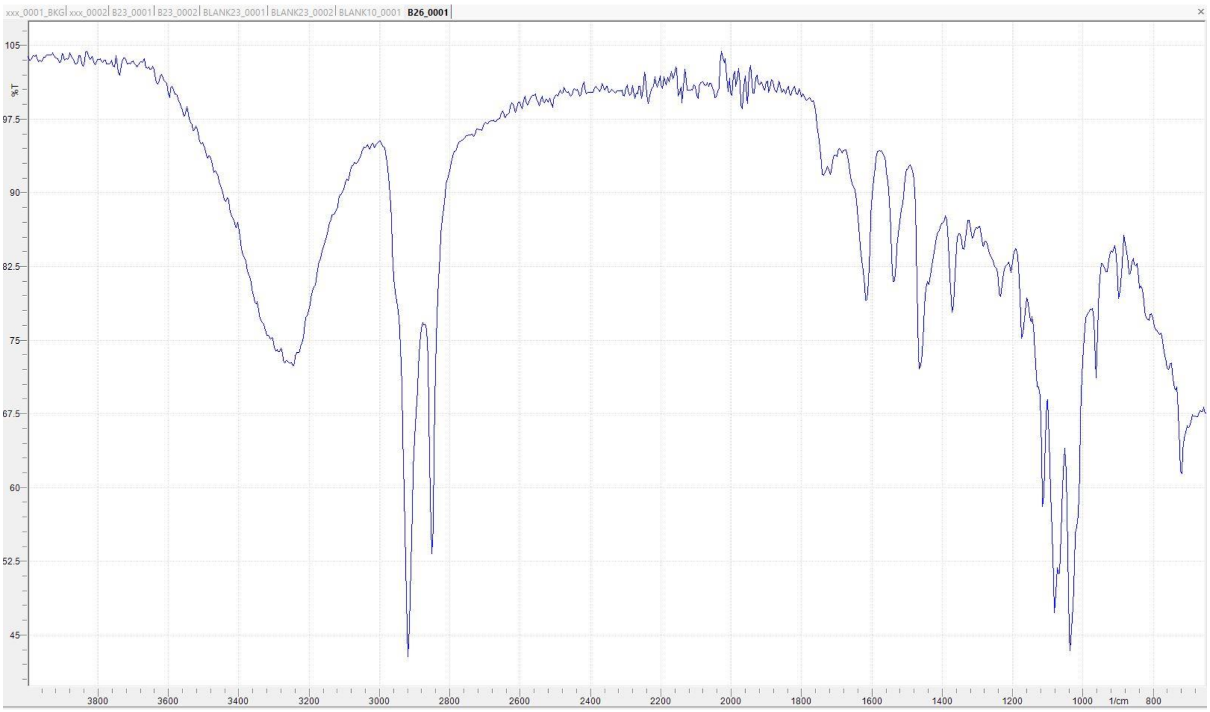

Figure S1. FT-IR spectrum of compound **1**

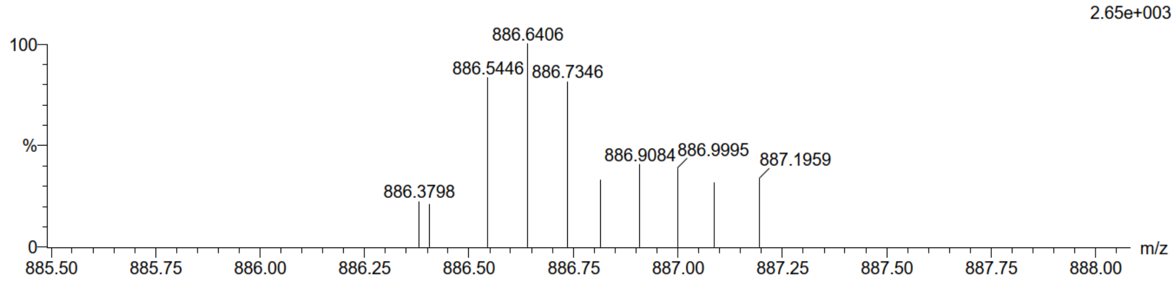

Minimum:				-1.5				
Maximum:		5.0	10.0	50.0				
Mass	Calc. Mass	mDa	PPM	DBE	i-FIT	Norm	Conf(%)	Formula
886.7346	886.7347	-0.1	-0.1	2.5	95.7	0.851	42.71	C51 H100 N O10
	886.7304	4.2	4.7	4.5	96.0	1.106	33.09	C4 O51 23Na
	886.7323	2.3	2.6	-0.5	96.3	1.419	24.20	C49 H101 N O10 23Na

Figure S2. HRESIMS(+) of compound **1**

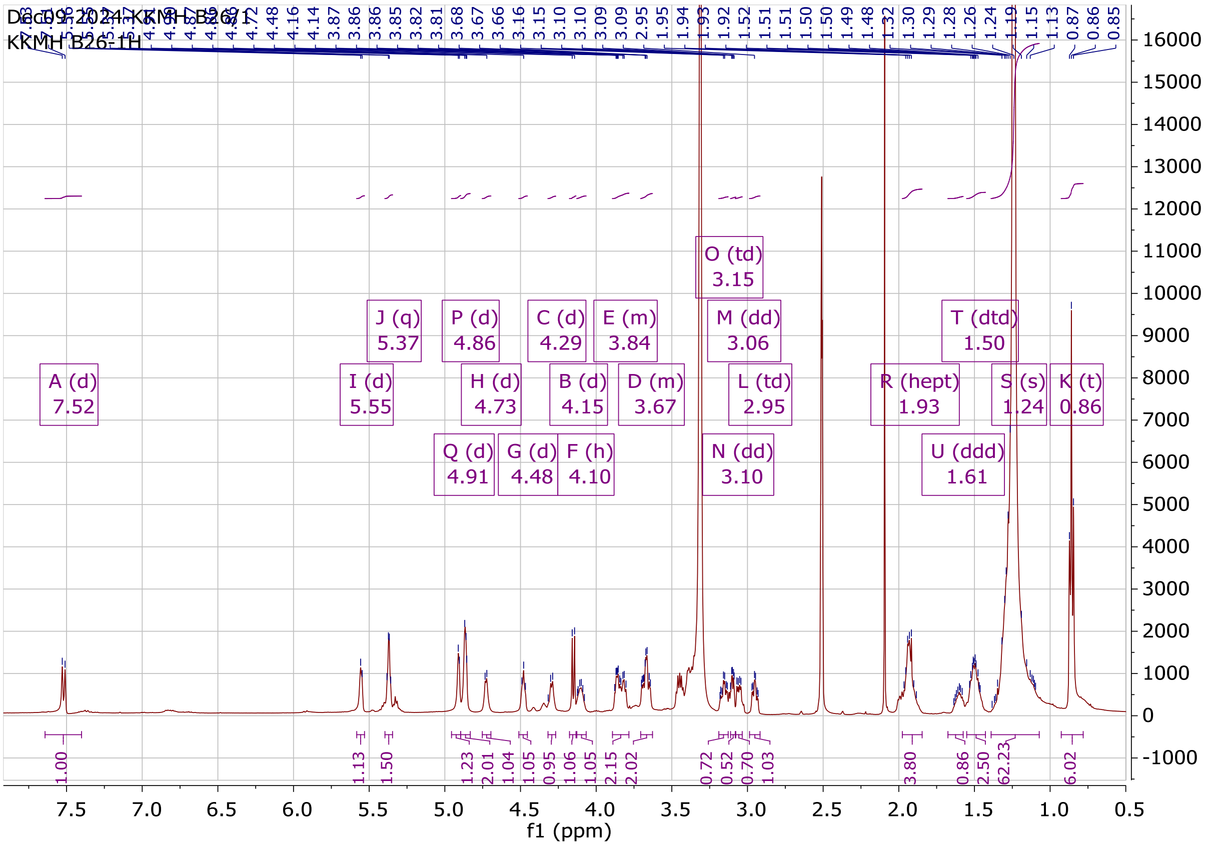

Figure S3. ^{1}H NMR (DMSO-d_6, 500 MHz) spectrum of compound **1**

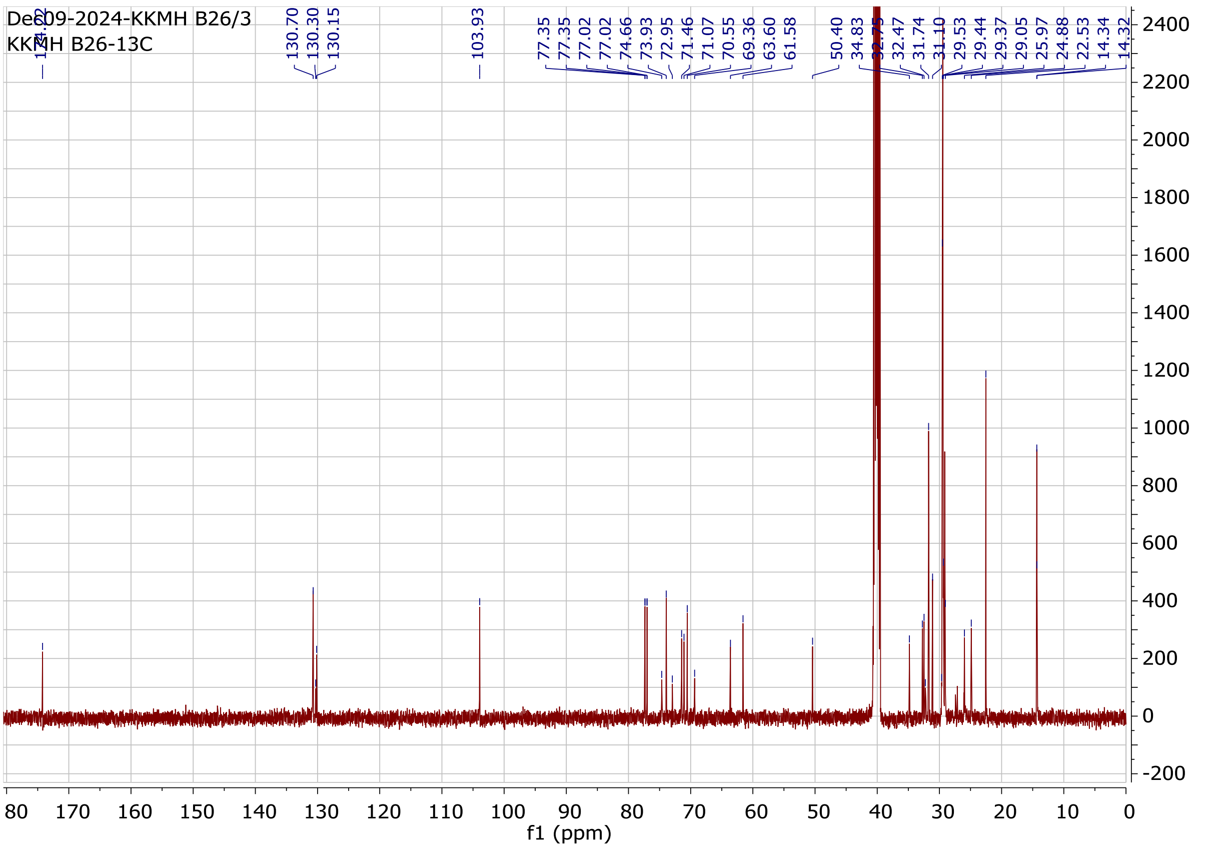

Figure S4. ^{13}C NMR (DMSO-d_6, 125 MHz) spectrum of compound **1**

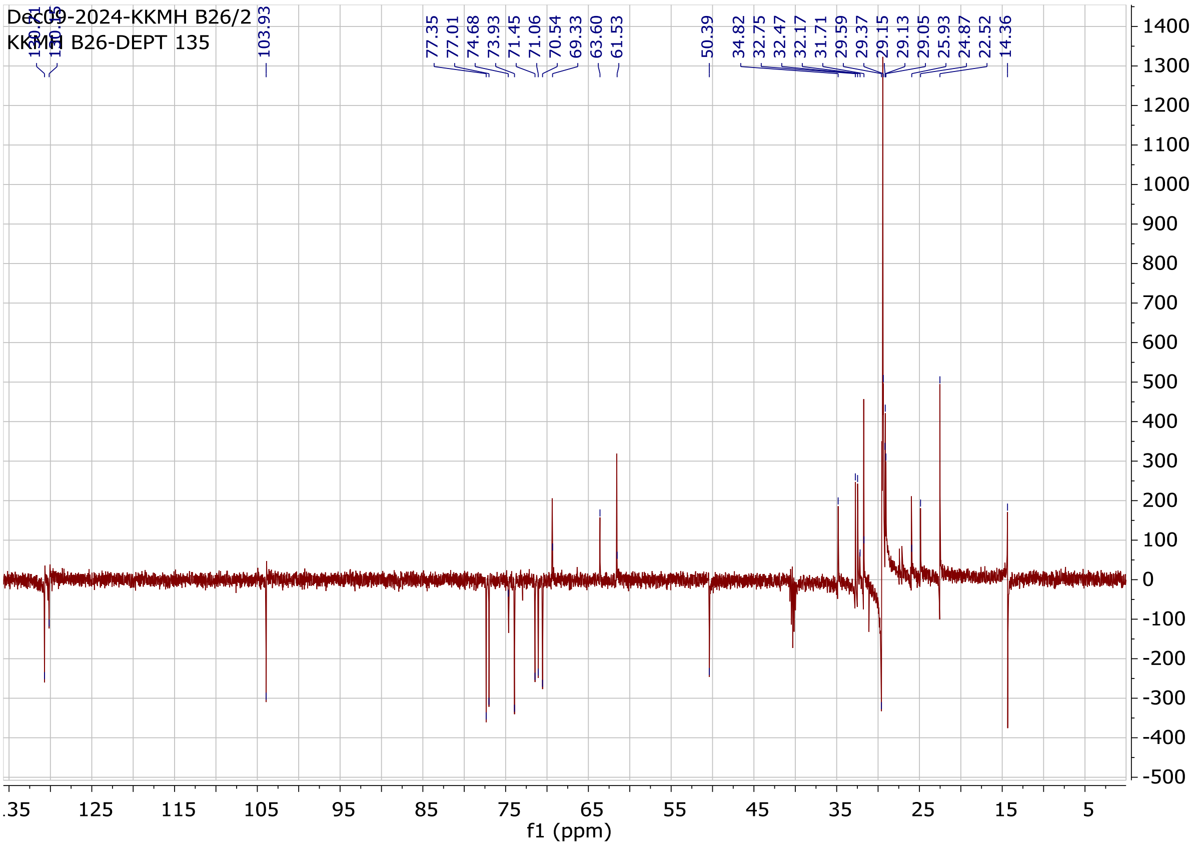

Figure S5. DEPT 135 (DMSO-d_6, 125 MHz) spectrum of compound **1**

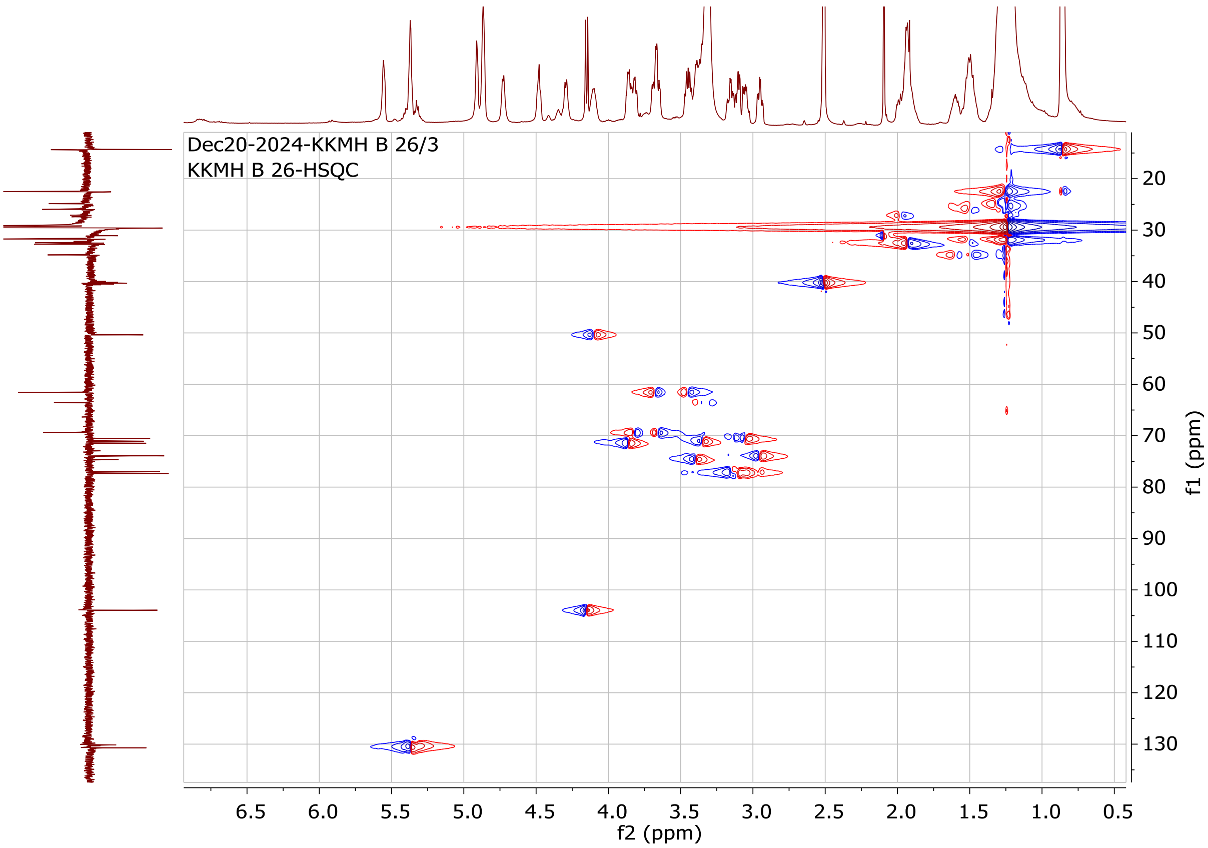

Figure S6. HSQC spectrum of compound **1**

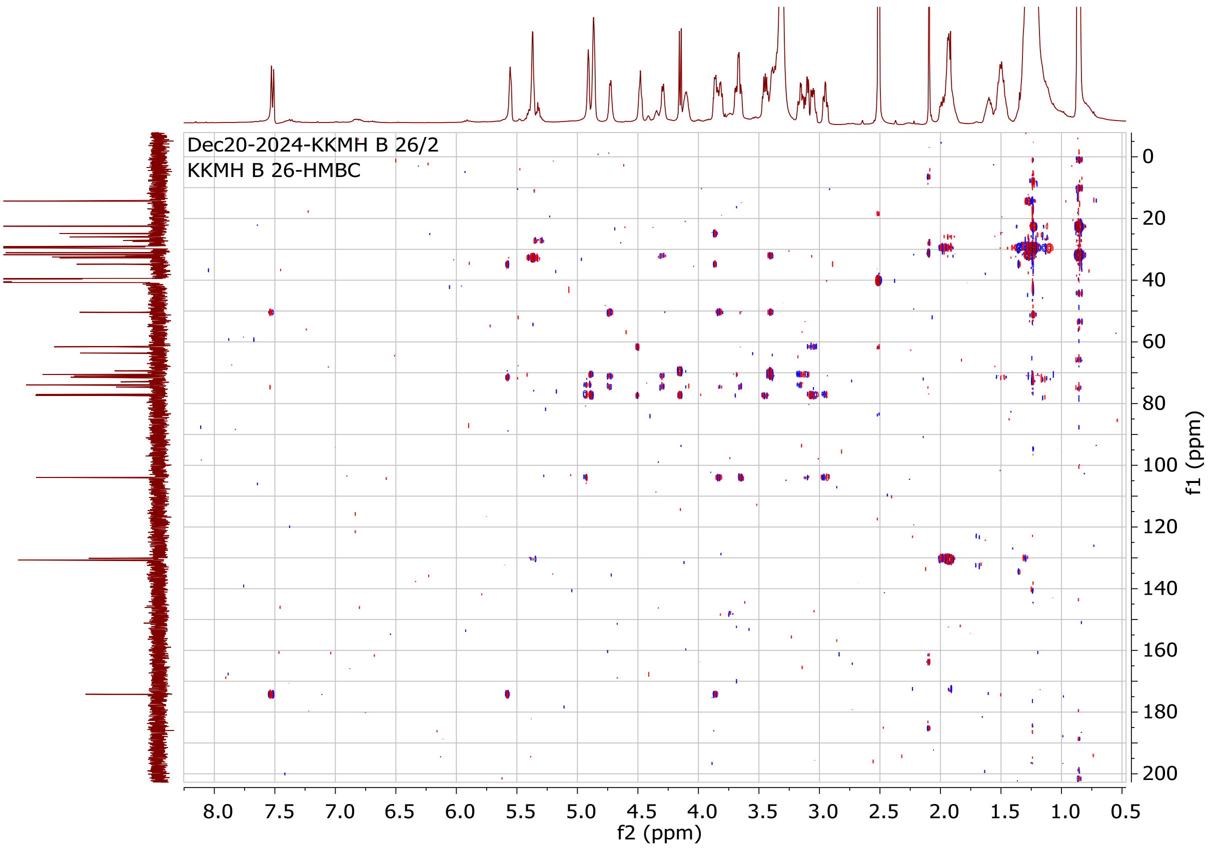

Figure S7. HMBC spectrum of compound **1**

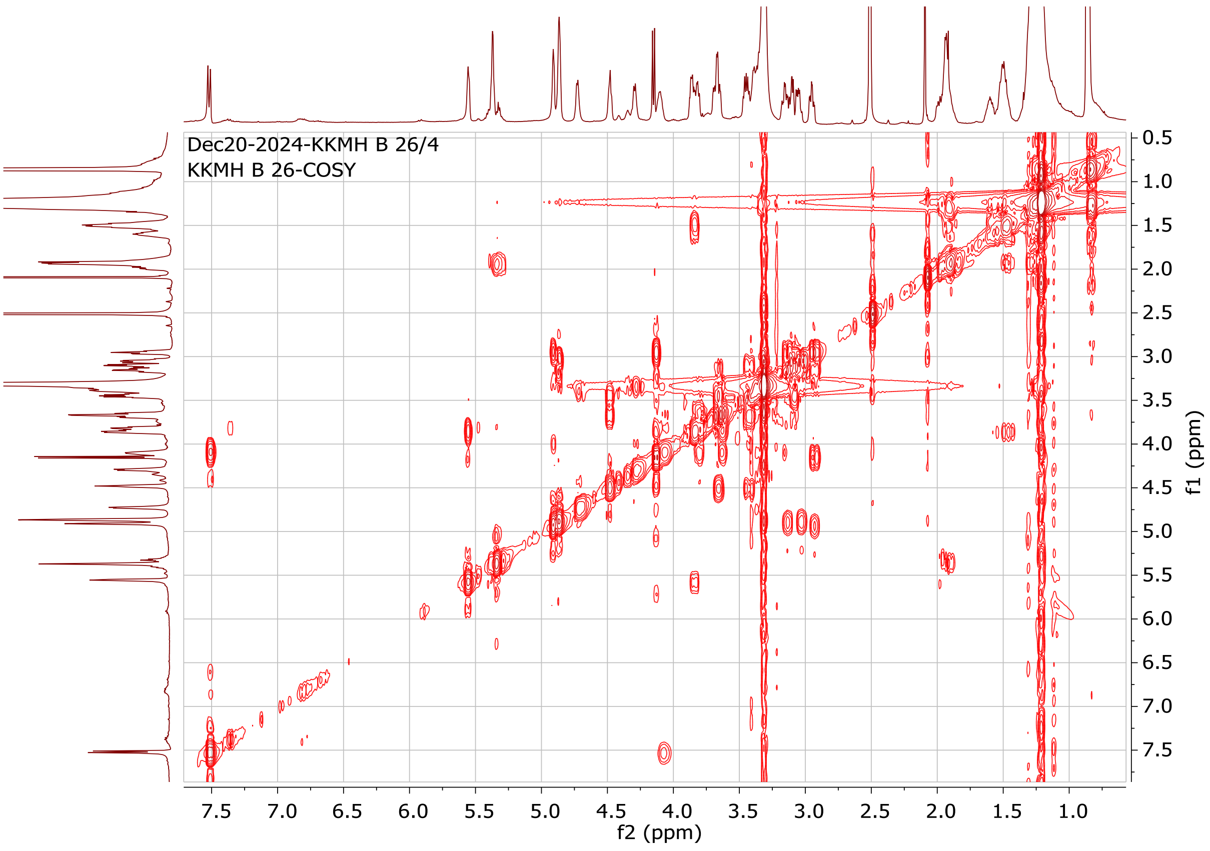

Figure S8. COSY spectrum of compound **1**

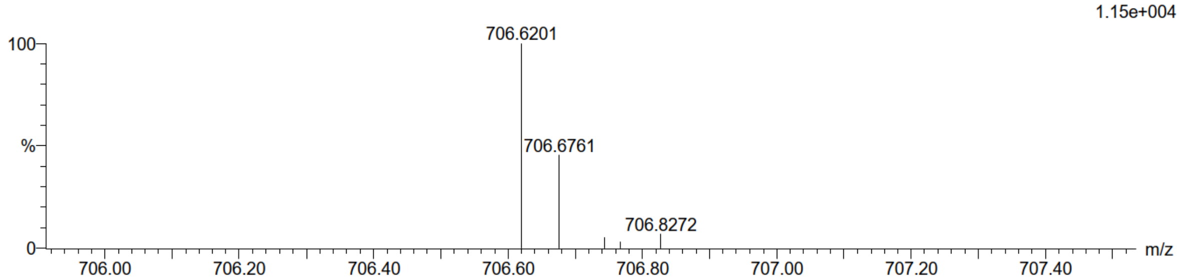

Minimum:				-1.5				
Maximum:		5.0	10.0	50.0				

Mass	Calc. Mass	mDa	PPM	DBE	i-FIT	Norm	Conf(%)	Formula
706.6761	706.6713	4.8	6.8	2.5	68.3	n/a	n/a	C45 H88 N O4

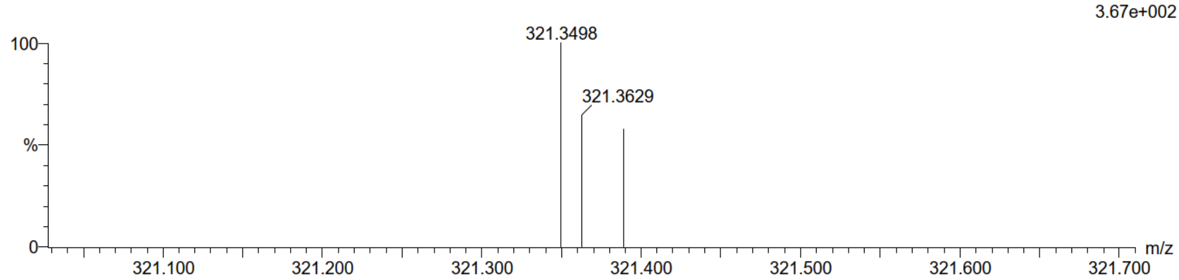

Minimum:				-1.5				
Maximum:		5.0	10.0	50.0				

Mass	Calc. Mass	mDa	PPM	DBE	i-FIT	Norm	Conf(%)	Formula
321.3498	321.3497	0.1	0.3	-1.5	42.7	0.380	68.37	C21 H46 23Na
	321.3521	-2.3	-7.2	1.5	43.5	1.151	31.63	C23 H45

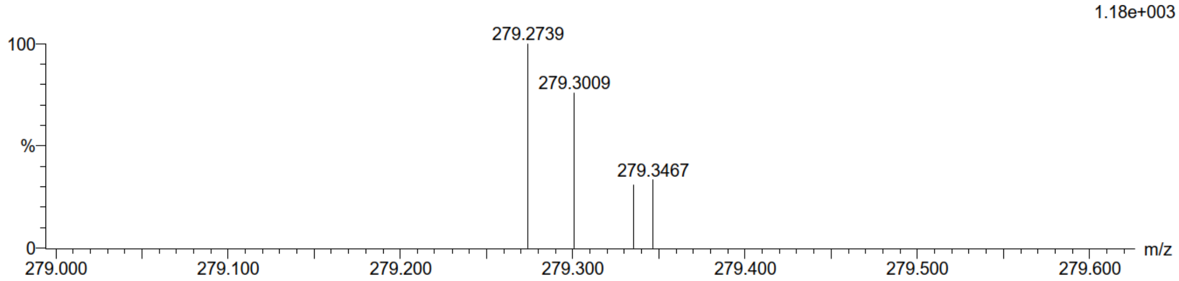

Minimum:				-1.5				
Maximum:		5.0	10.0	50.0				

Mass	Calc. Mass	mDa	PPM	DBE	i-FIT	Norm	Conf(%)	Formula
279.3009	279.3028	-1.9	-6.8	-1.5	47.2	0.234	79.17	C18 H40 23Na
	279.3052	-4.3	-15.4	1.5	48.6	1.569	20.83	C20 H39

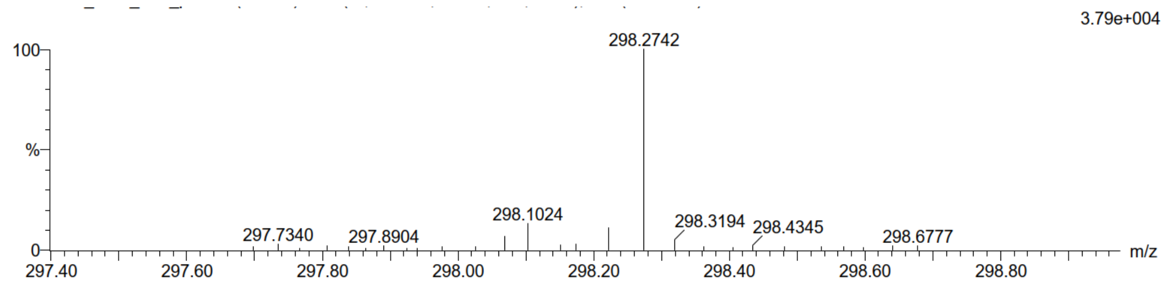

	Mass	Calc. Mass	mDa	PPM	DBE	i-FIT	Norm	Conf(%)	Formula
Minimum:					-1.5				
Maximum:			5.0	10.0	50.0				
	298.2742	298.2746	-0.4	-1.3	1.5	161.2	0.493	61.08	C18 H36 N O2
		298.2722	2.0	6.7	-1.5	161.7	0.944	38.92	C16 H37 N O2 23Na

Figure S9. HRESIMS(+) fragments of compound **1**

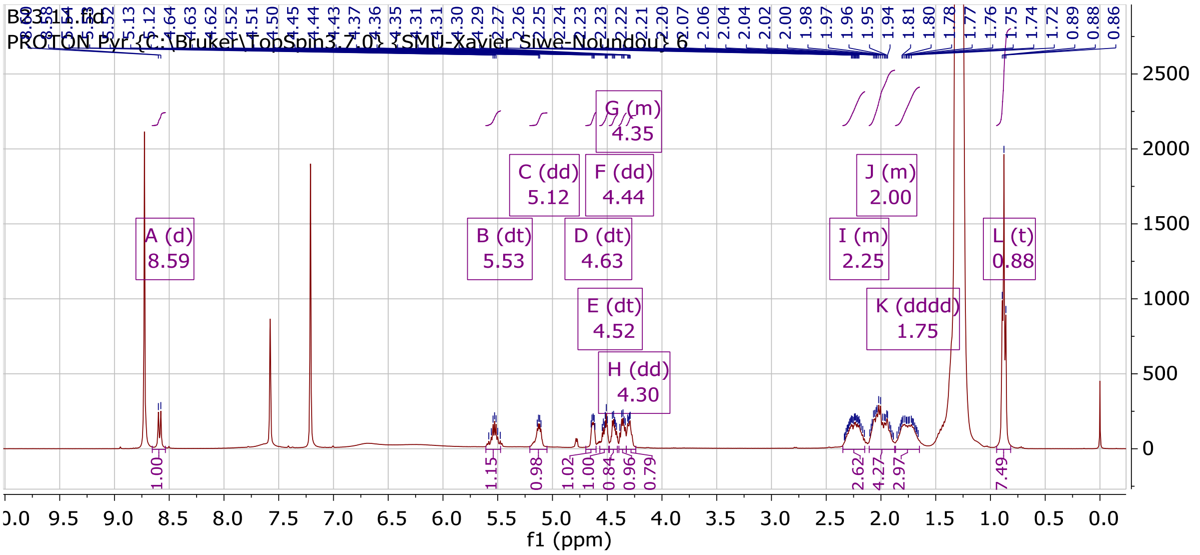

Figure S10. ^{1}H NMR (C_5D_5N, 400 MHz) spectrum of compound **2**

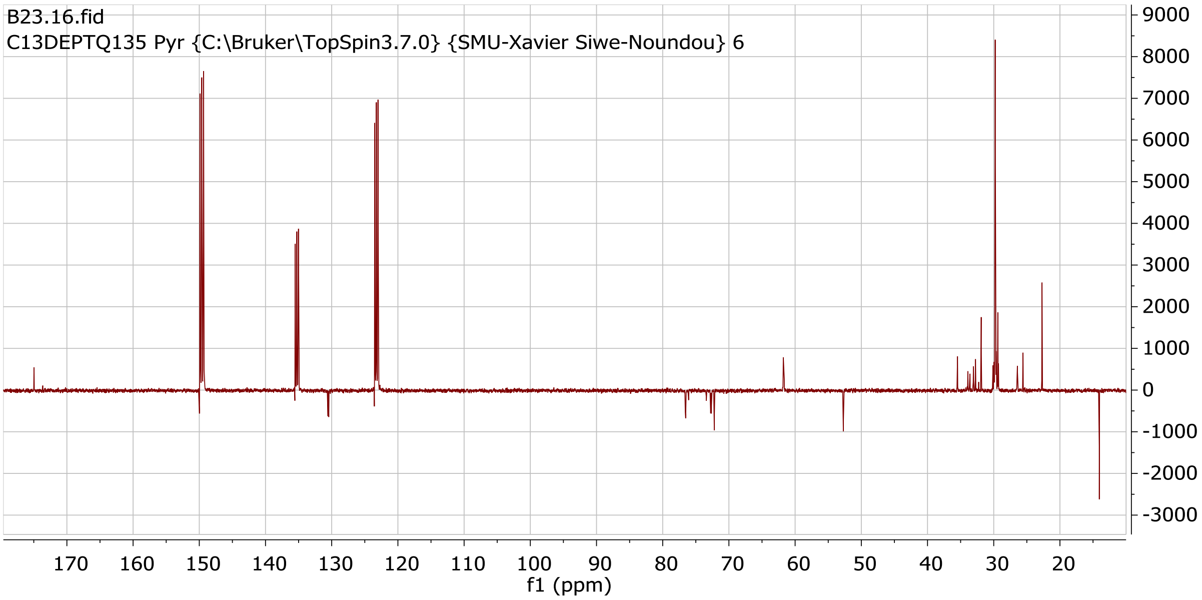

Figure S11. ^{13}C NMR (C_5D_5N, 100 MHz) spectrum of compound **2**

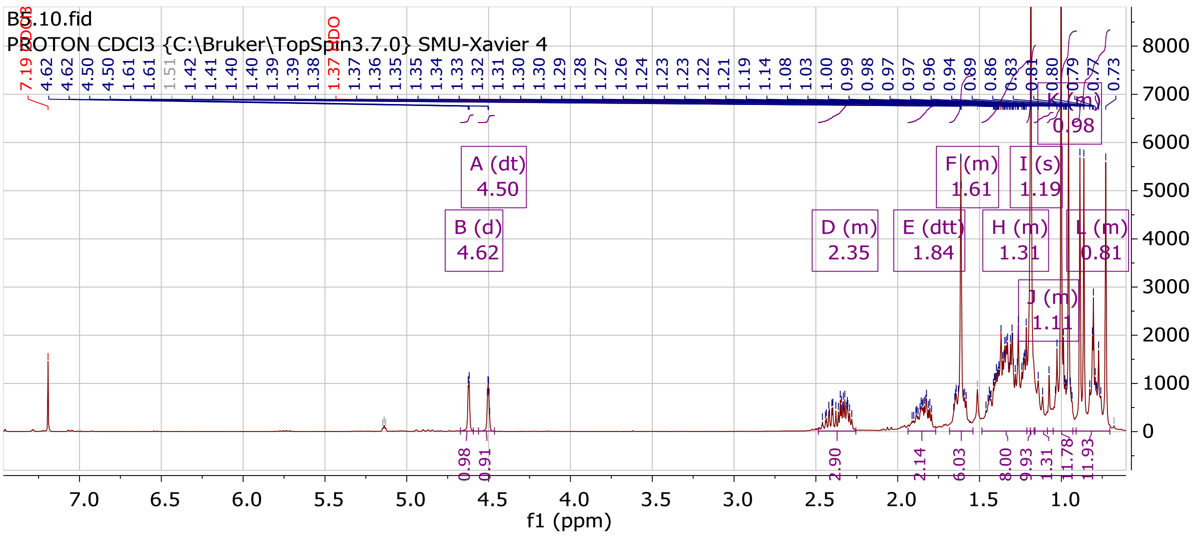

Figure S12a. ^{1}H NMR ($CDCl_3$, 400 MHz) spectrum of compound **3**

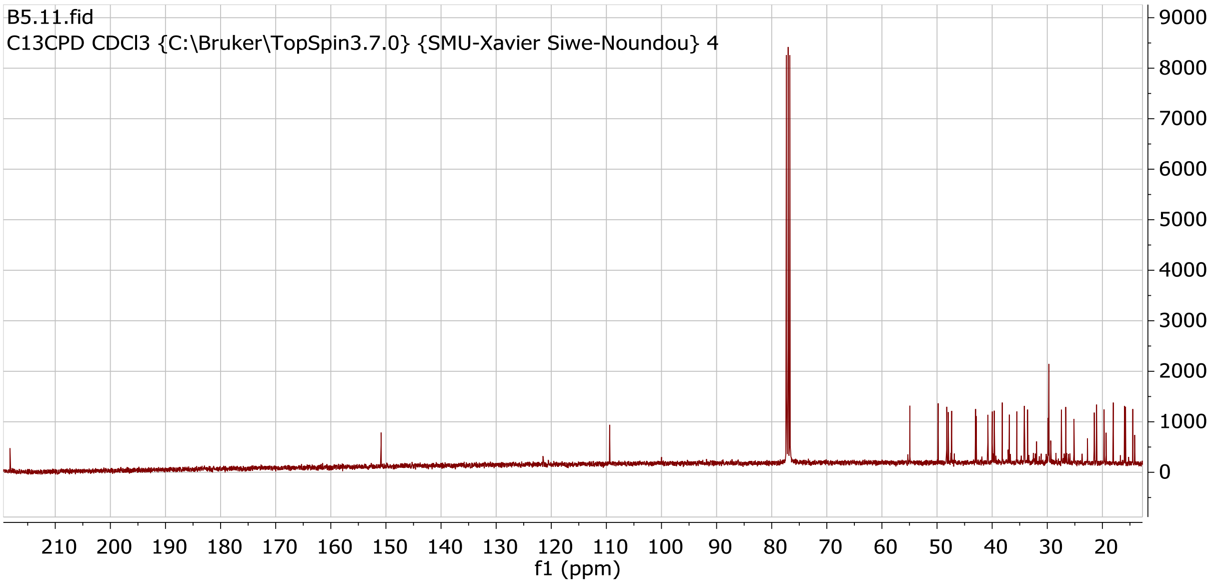

Figure S12b. ^{13}C NMR ($CDCl_3$, 100 MHz) spectrum of compound **3**

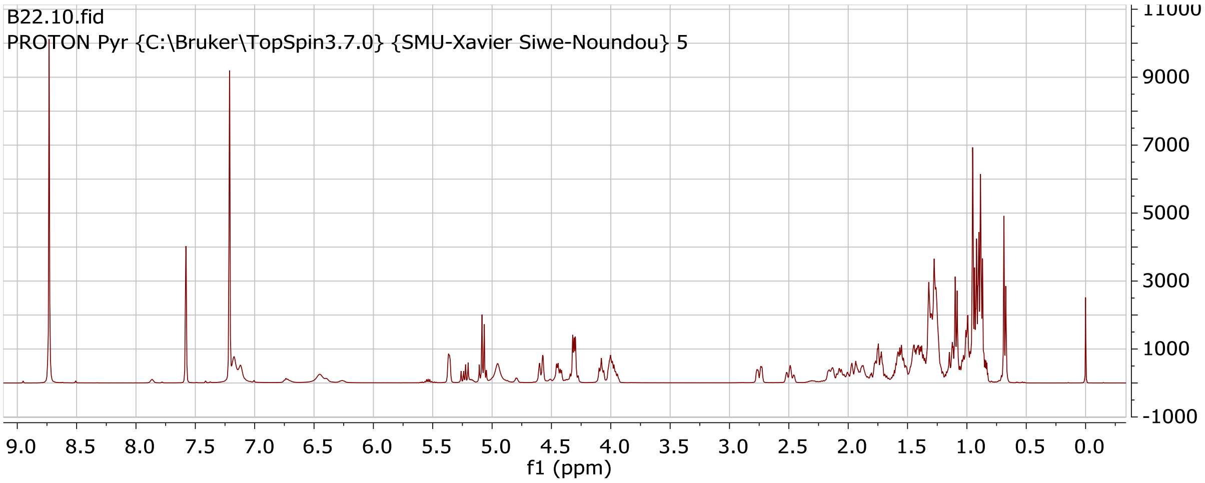

Figure S13a. ^{1}H NMR (C_5D_5N, 500 MHz) spectrum of compound **4**

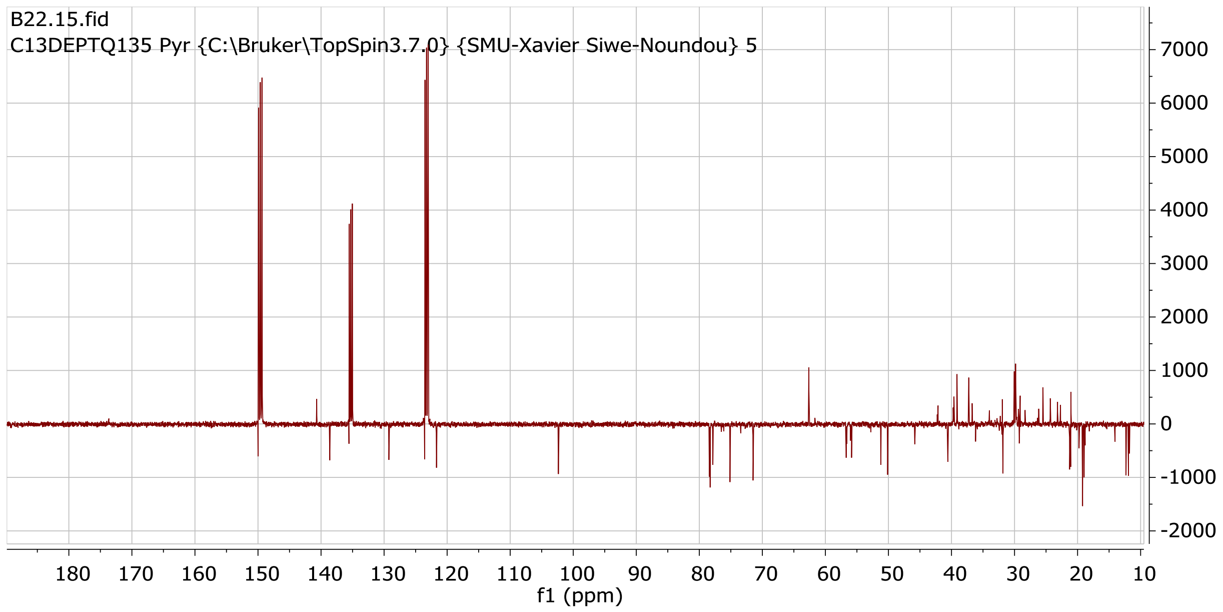

Figure S13b. ^{13}C NMR ($CDCl_3$, 400 MHz) spectrum of compound **4**

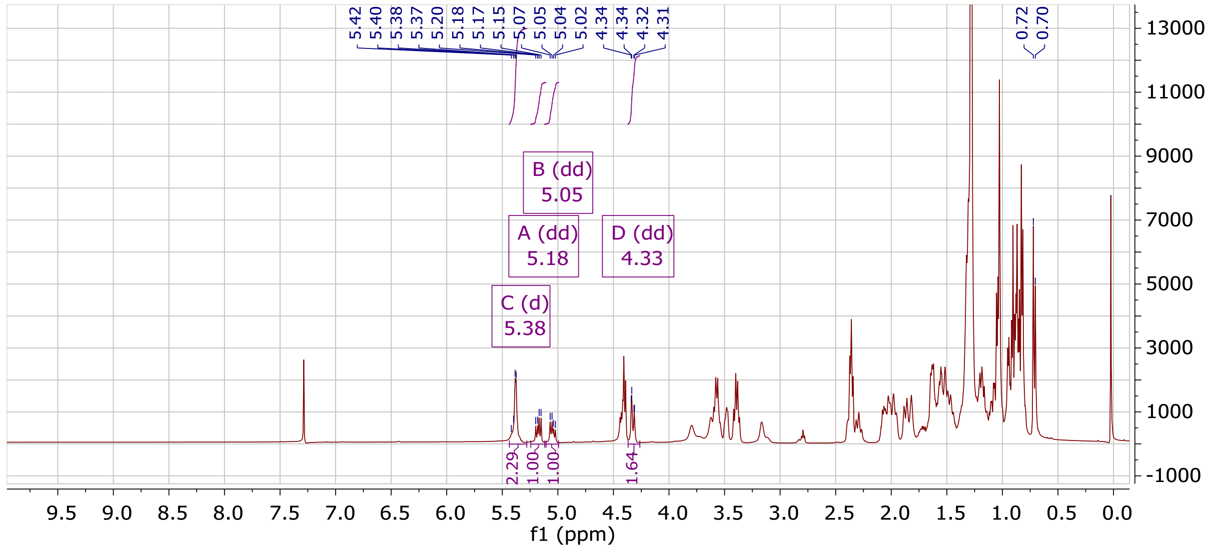

Figure S14a. ^{1}H NMR ($CDCl_3$, 400 MHz) spectrum of compound **5**

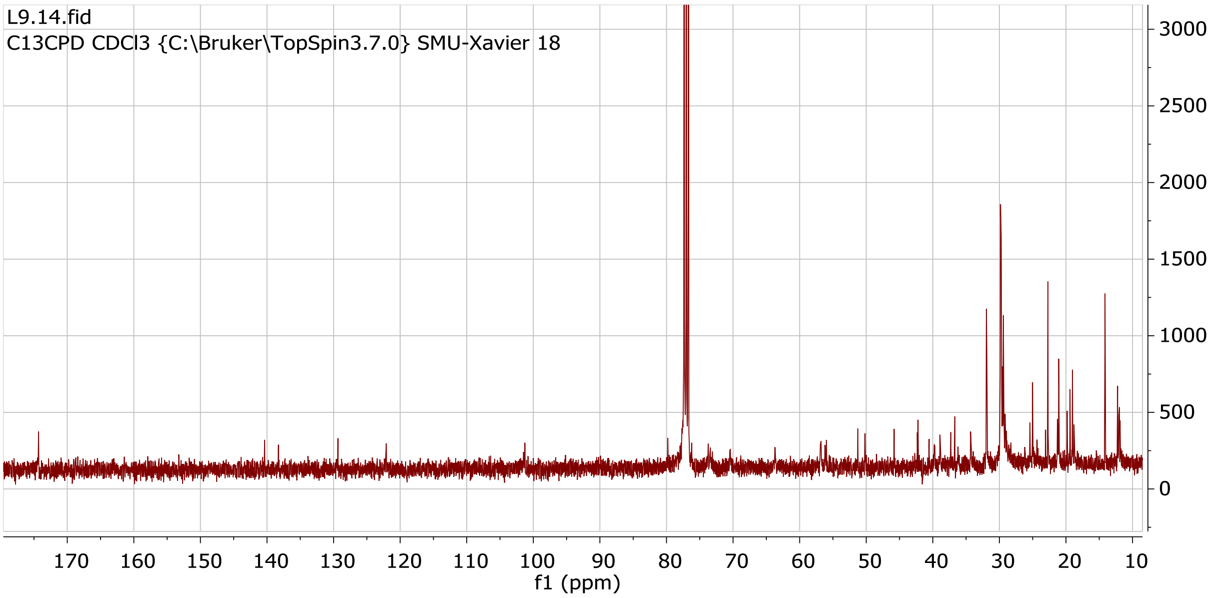

Figure S14b. ^{13}C NMR ($CDCl_3$, 100 MHz) spectrum of compound **5**

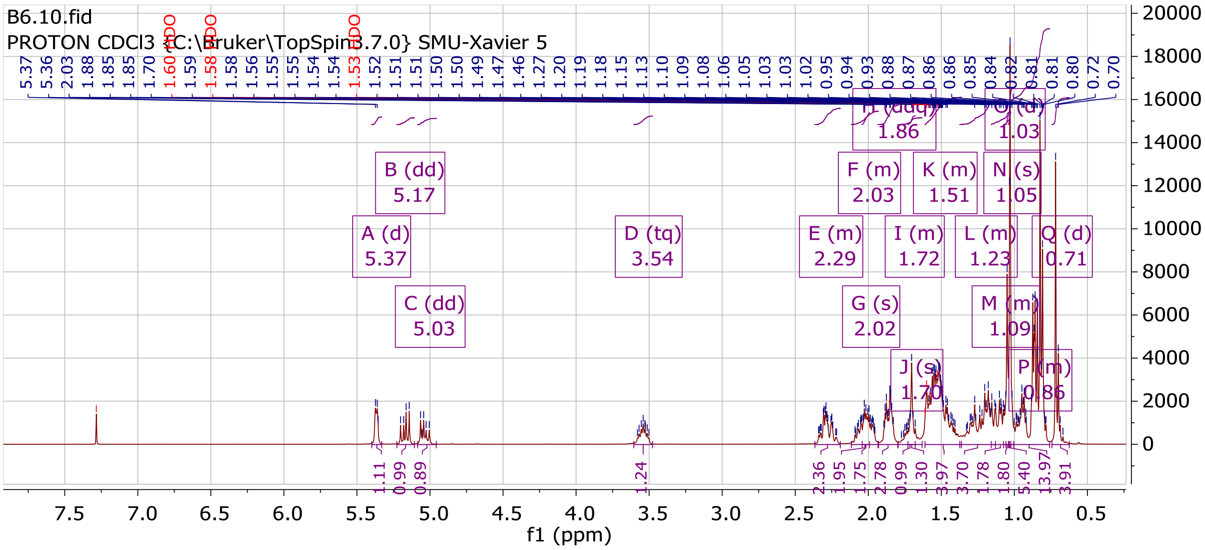

Figure S15a. ^{1}H NMR ($CDCl_3$, 400 MHz) spectrum of compound **6**

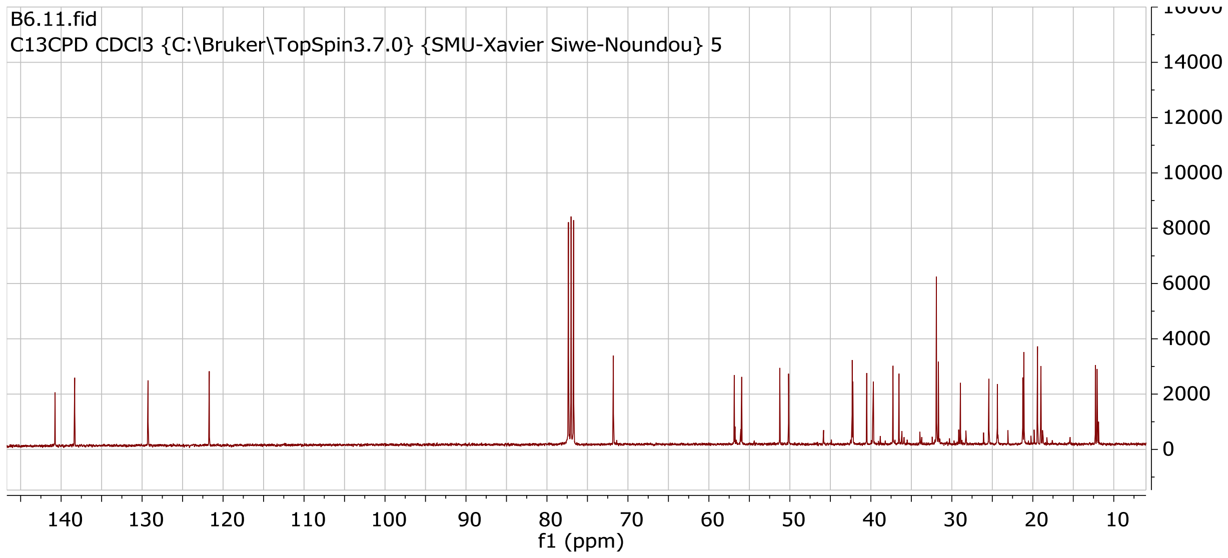

Figure S15b. ^{13}C NMR ($CDCl_3$, 100 MHz) spectrum of compound **6**

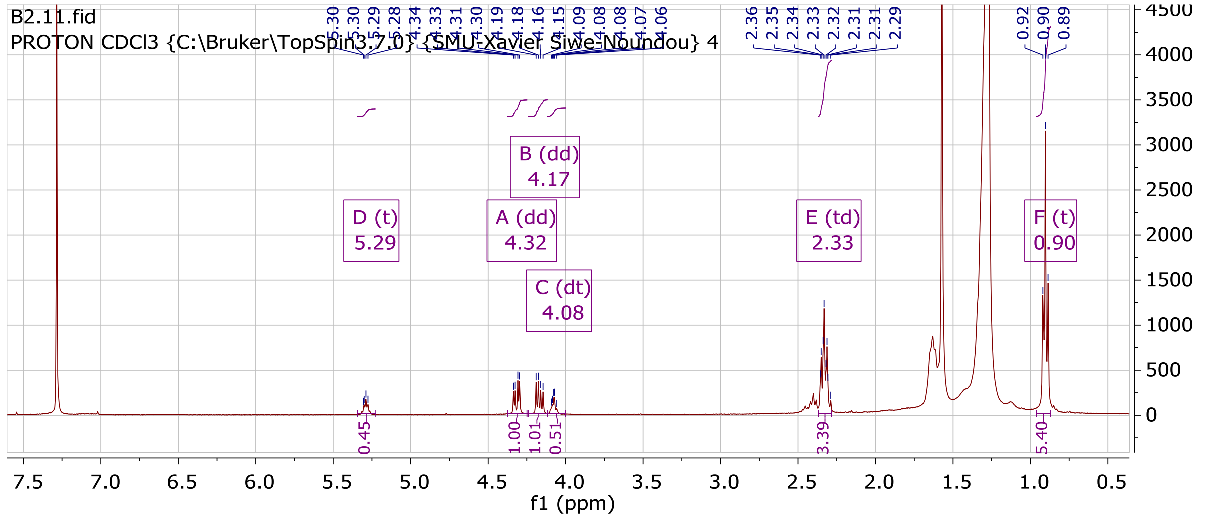

Figure S16a. ^{1}H NMR ($CDCl_3$, 400 MHz) spectrum of compound **7**

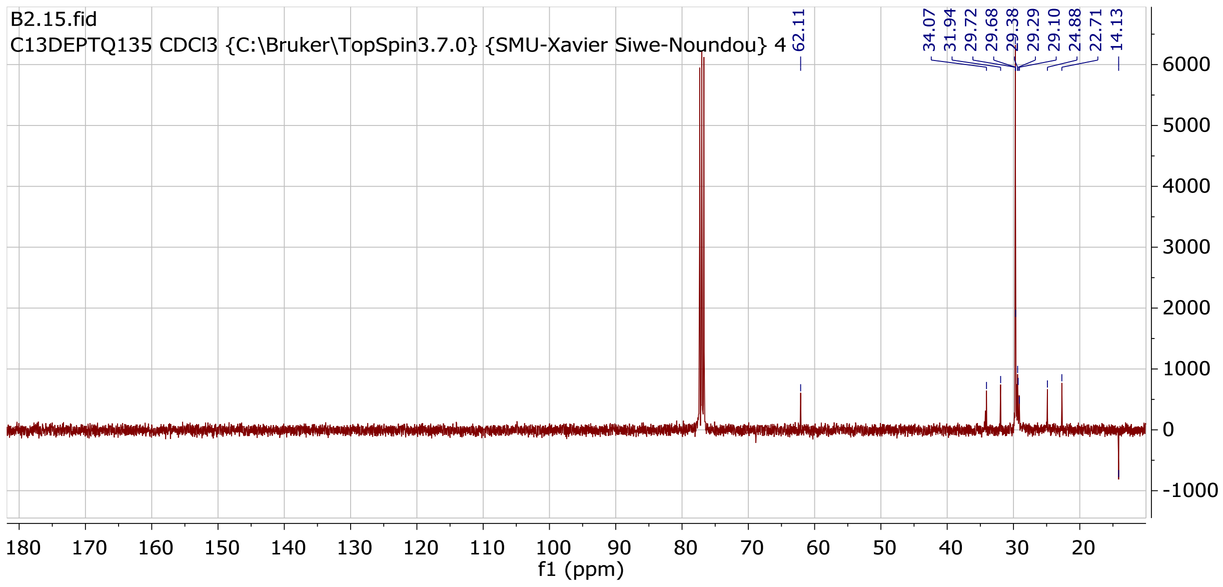

Figure S16b. ^{13}C NMR ($CDCl_3$, 100 MHz) spectrum of **7**

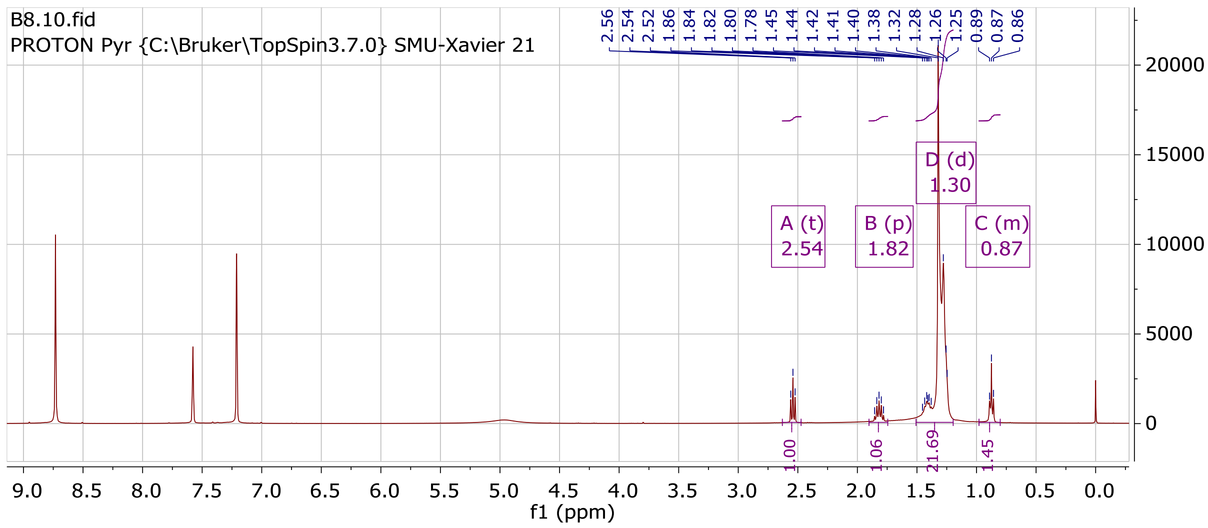

Figure S17a. ^{1}H NMR (CD_5N, 400 MHz) spectrum of compound **8**

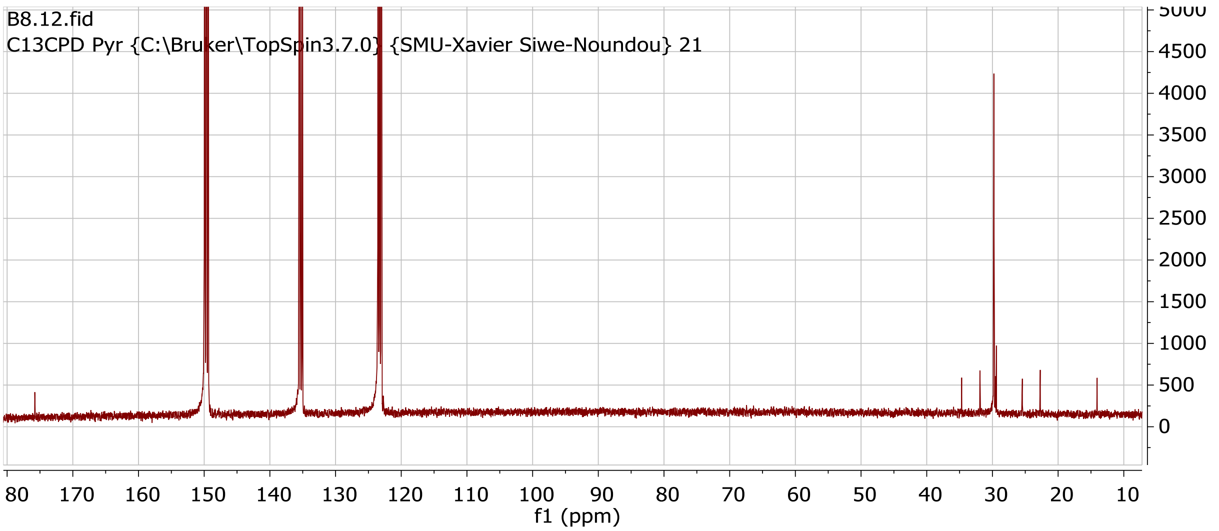

Figure S17b. ^{13}C NMR (C_5D_5N, 100 MHz) spectrum of **8**

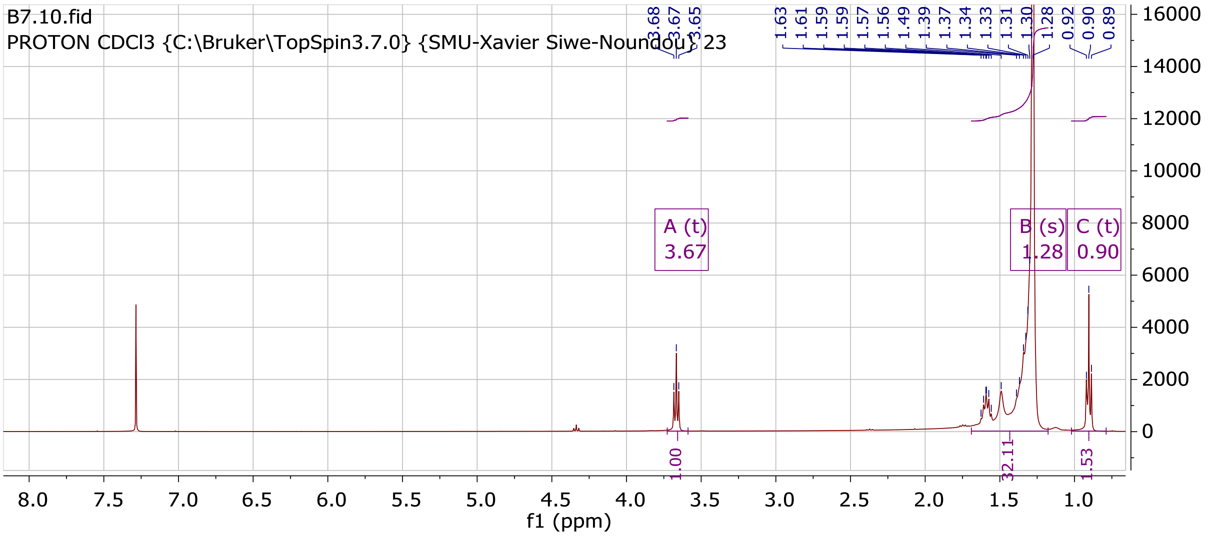

Figure S18a. ^{1}H NMR ($CDCl_3$, 400 MHz) spectrum of compound **9**

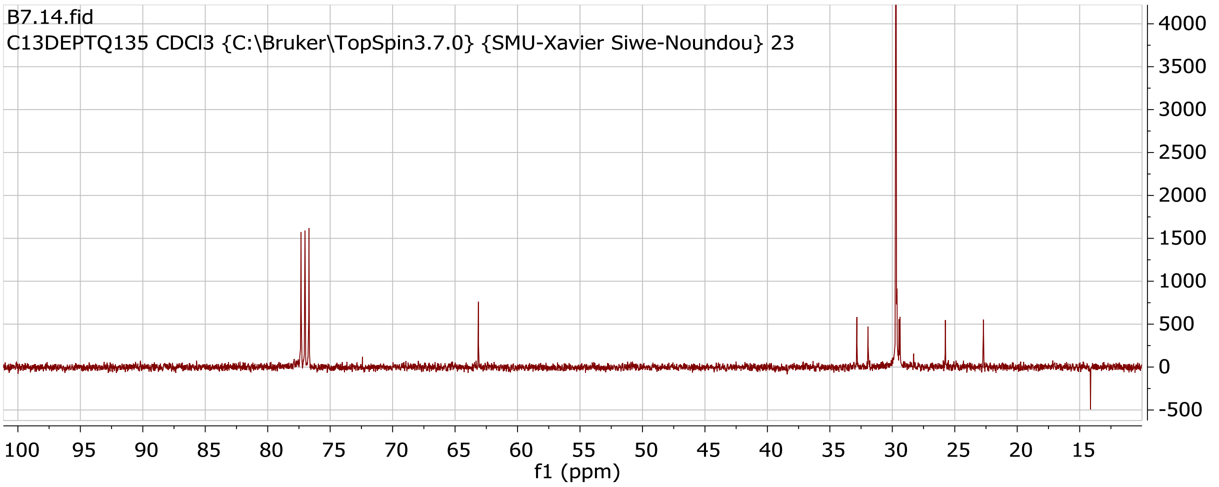

Figure S19b. ^{13}C NMR ($CDCl_3$, 100 MHz) spectrum of **9**

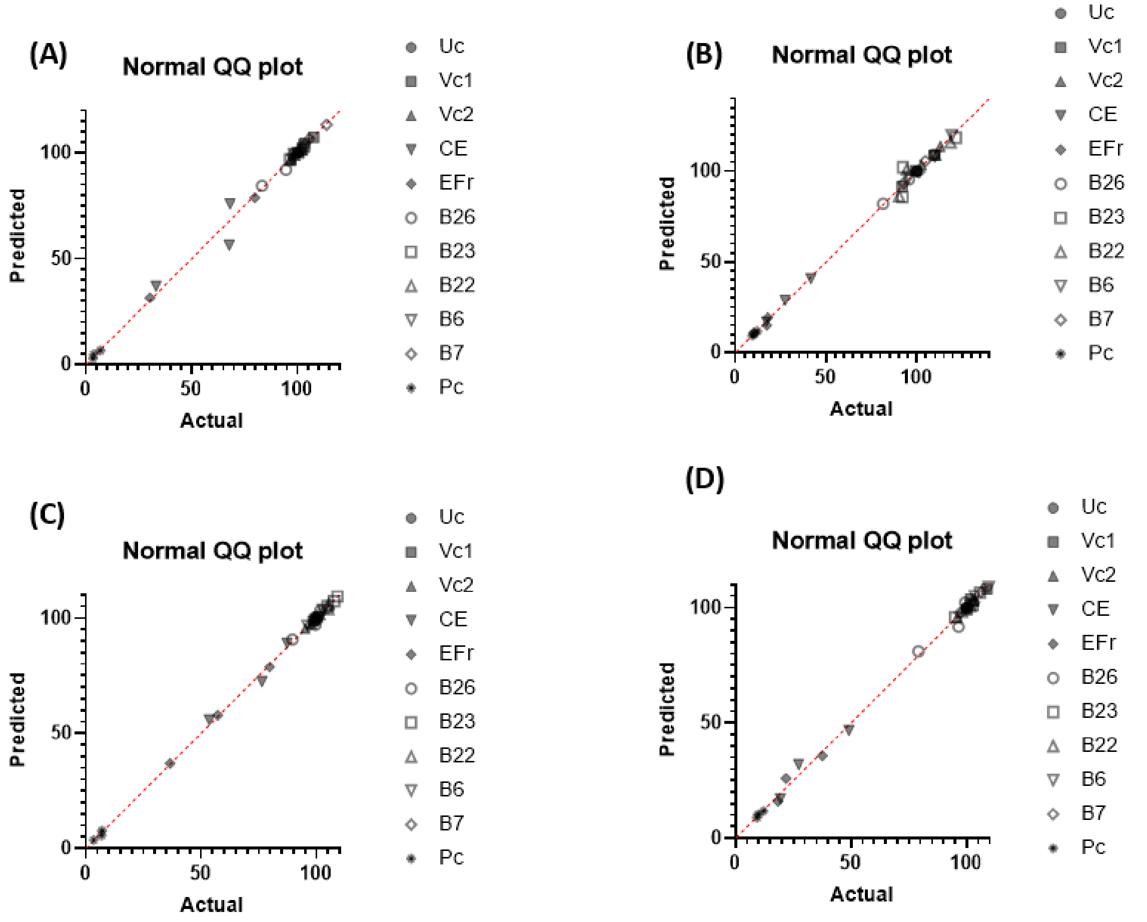

(A)
Normal QQ plot
Predicted
Actual
0
50
100
Uc
Vc1
Vc2
CE
EFr
B26
B23
B22
B6
B7
Pc
(B)
Normal QQ plot
Predicted
Actual
0
50
100
Uc
Vc1
Vc2
CE
EFr
B26
B23
B22
B6
B7
Pc
(C)
Normal QQ plot
Predicted
Actual
0
50
100
Uc
Vc1
Vc2
CE
EFr
B26
B23
B22
B6
B7
Pc
(D)
Normal QQ plot
Predicted
Actual
0
50
100
Uc
Vc1
Vc2
CE
EFr
B26
B23
B22
B6
B7
Pc

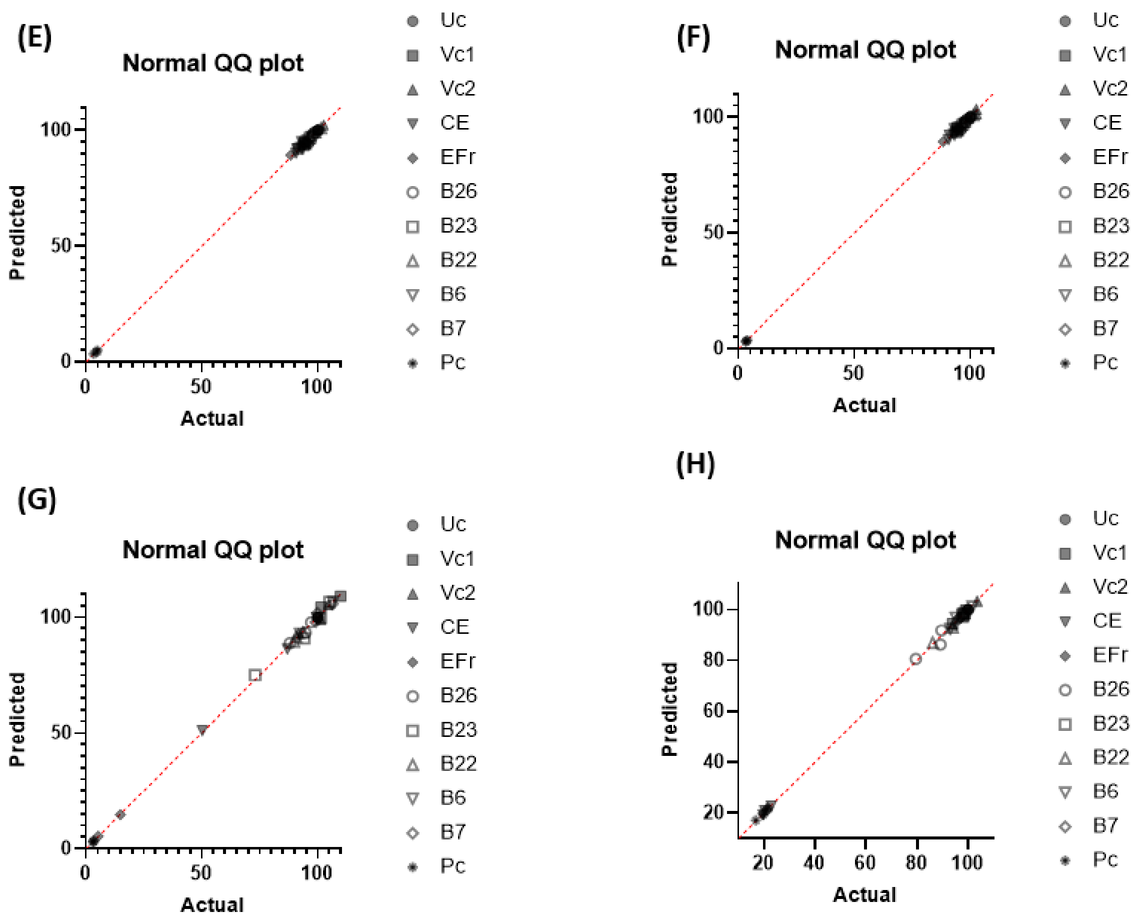

Figure S19. Normality graphs in Shapiro-Wilk test, A = HaCaT resazurin assay, B = HaCaT CV assay, C = NHEM-Ad Resazurin assay, D = NHEM-Ad CV assay, E = HepG2 resazurin assay, F = HepG2 CV assay, G = HEK 293 resazurin assay, and H = HEK 293 CV assay

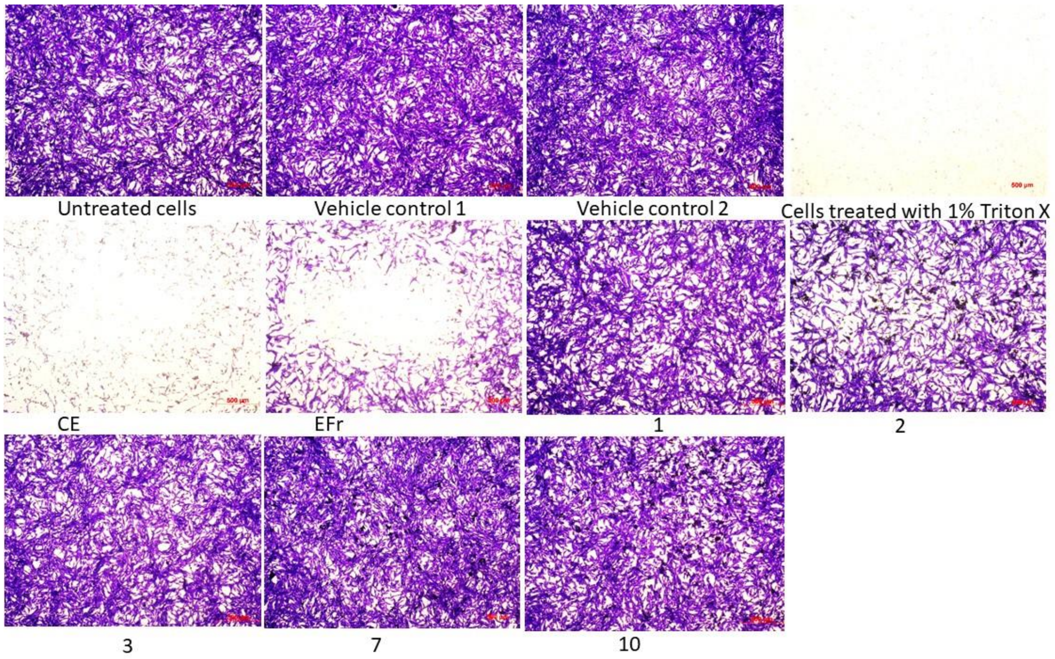

Figure S20. Microscopic images of the wells for HaCaT cells

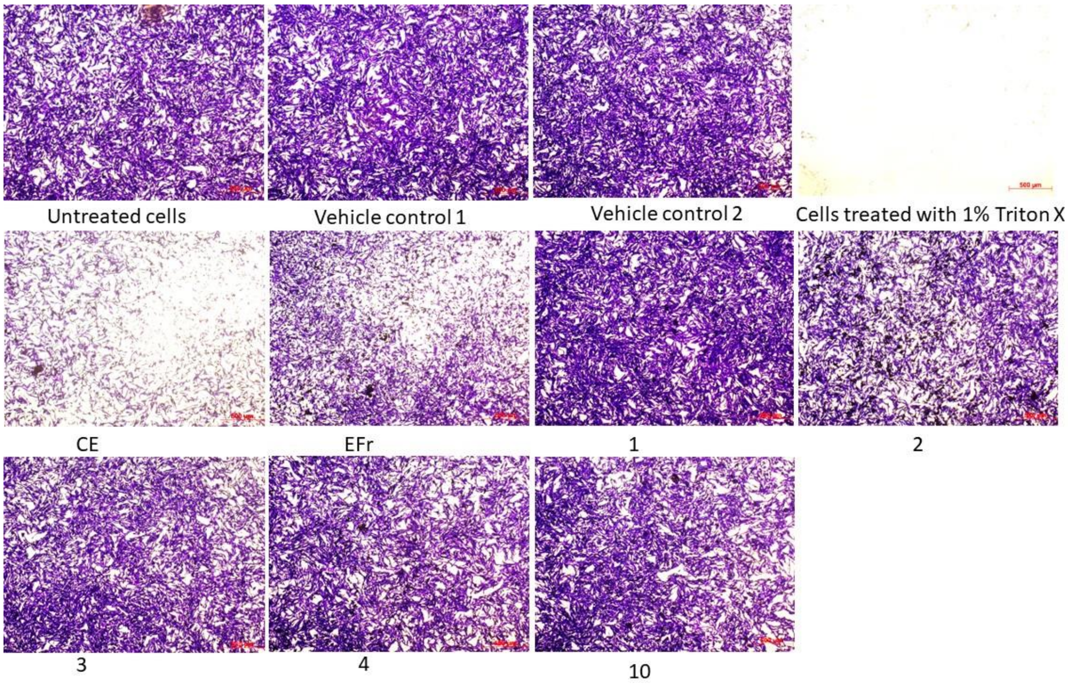

Figure S21. Microscopic images of the wells for NHEM-Ad cells
